# A Pan-GTPase Inhibitor as a Molecular Probe

**DOI:** 10.1371/journal.pone.0134317

**Published:** 2015-08-06

**Authors:** Lin Hong, Yuna Guo, Soumik BasuRay, Jacob O. Agola, Elsa Romero, Denise S. Simpson, Chad E. Schroeder, Peter Simons, Anna Waller, Matthew Garcia, Mark Carter, Oleg Ursu, Kristine Gouveia, Jennifer E. Golden, Jeffrey Aubé, Angela Wandinger-Ness, Larry A. Sklar

**Affiliations:** 1 Department of Pathology, University of New Mexico, Albuquerque, New Mexico, United States of America; 2 University of New Mexico Center for Molecular Discovery, Albuquerque, New Mexico, United States of America; 3 Cancer Research and Treatment Center, University of New Mexico, Albuquerque, New Mexico, United States of America; 4 Department of Biochemistry and Molecular Biology, University of New Mexico, Albuquerque, New Mexico, United States of America; 5 University of Kansas Specialized Chemistry Center, Lawrence, Kansas, United States of America; 6 Department of Medicinal Chemistry, University of Kansas, Lawrence, Kansas, United States of America; University of Lethbridge, CANADA

## Abstract

Overactive GTPases have often been linked to human diseases. The available inhibitors are limited and have not progressed far in clinical trials. We report here a first-in-class small molecule pan-GTPase inhibitor discovered from a high throughput screening campaign. The compound CID1067700 inhibits multiple GTPases in biochemical, cellular protein and protein interaction, as well as cellular functional assays. In the biochemical and protein interaction assays, representative GTPases from Rho, Ras, and Rab, the three most generic subfamilies of the GTPases, were probed, while in the functional assays, physiological processes regulated by each of the three subfamilies of the GTPases were examined. The chemical functionalities essential for the activity of the compound were identified through structural derivatization. The compound is validated as a useful molecular probe upon which GTPase-targeting inhibitors with drug potentials might be developed.

## Introduction

The Ras superfamily GTPases are comprised of about 150 small monomeric guanine nucleotide binding proteins. The small GTPases can be grouped into several subfamilies based on sequence similarities and functional specifications. Three generic subfamilies include the Rho, Rab and Ras GTPases: Rho GTPases regulate cytoskeletal organization and cell cycle progression with Rho, Rac and Cdc42 being representative members [1, 2]; Rab GTPases play roles in membrane trafficking and molecular cargo degradation [3, 4]; Ras GTPases are signal transduction regulators modulating multiple essential molecular pathways [5, 6]. The intrinsic hydrolytic activity of GTPases converts the associated GTP to GDP and regulates concomitant conformational changes from an active to inactive state. The functions of GTPases are locally and temporally controlled through interactions with other proteins including GTPase-activating proteins (GAPs) that enhance nucleotide hydrolysis, the guanine nucleotide exchange factors (GEFs) that facilitate nucleotide exchange, GTPase prenyl- and farnesyltransferases that regulate membrane localization, and effectors that lead to downstream signaling.

Mutations and aberrant gene expression of GTPases have been associated with human diseases including cancers, immunodeficiency diseases, and neurological disorders [7–10]. Significantly, hyperactive Ras has been found in about a third of human carcinomas [11, 12]. Therefore the search for GTPase inhibitors has spanned several decades. The earliest inhibitors acted through inhibiting the lipid transferases which modify GTPases for membrane localization and subsequent activation. [13]. However, the toxicities associated with inhibiting the lipid transferases thwarted their usefulness. Accumulating biochemical and structural studies showed that the GTPases are difficult drug targets because of their high ligand affinity and their small globular nature which makes it difficult to locate a drug binding pocket [9, 14]. However, considerable progress has been made when structural information especially that of the complexes formed between GTPases and their regulators and effectors, is available. *In silico* virtual screening and docking has enabled identification and development of Ras, Rho and Rac inhibitors that block the interactions between the GTPase and its GEF or effector [15–17]. From the crystal structures of Rab in complex with protein binding partners, peptides stabilized by hydrocarbon stapling and bound to Rab GTPases were developed. One peptide StRIP3 selectively bound to activated Rab8a and inhibited a Rab8a-effector interaction [18]. Biochemical screening yielded a Cdc42 selective inhibitor that abolishes nucleotide binding and blocks the cellular functions of Cdc42 [19]. A small molecule interfering with the interactions between the farnesylated K-Ras and prenyl-binding protein PDEδ was also discovered from screening and shown to inhibit oncogenic Ras signaling [20]. Some inhibitors have been developed to directly target the catalytic activity of GTPase GEFs and prevent the activation of their substrate GTPases [21, 22]. Efforts from chemical synthesis generated a metal complex that specifically targets activated Ras and a molecule that covalently labels the guanine nucleotide binding site of the oncogenic K-Ras G12C mutant [23–25]. Additional K-Ras G12C inhibitors were also developed that bound to an allosteric site beneath the switch-II region and blocked the effector interactions [26]. These small molecule compounds have served as important tools to inhibit individual GTPases in molecular studies. However, they have not had significant impact on disease management. Also, more versatile inhibitors that act against multiple GTPases can be useful when the GTPase activities need to be broadly blocked to dissect complicated molecular pathways.

Here we report the further characterization of a small molecule discovered from high throughput screening of the Molecular Libraries Small Molecule Repository (MLSMR) [27]. Previous biochemical studies have shown that compound CID1067700 (referred to as compound **1** hereafter) is a Rab7 inhibitor [28]. Here it is demonstrated that the compound can inhibit multiple GTPases when characterized in various biochemical assays and also shows inhibition efficacy in cellular analysis. The compound blocks guanine nucleotide binding to the GTPases. Though primarily a competitive inhibitor, the compound deviates from classical competitive behavior for some GTPases. This suggests the compound may have different binding modes towards different GTPases.

## Materials and Methods

GST-tagged GTPases were either from Cytoskeleton or purified as described previously [29]. Cyto-Plex microspheres (4.0 μm) were from Thermo Fisher Scientific. BODIPY^-^FL 2’-(or-3’)-*O*-(*N*-(2-aminoethyl) urethane) GTP and BODIPY^-^FL GDP were from Life Technologies. Alexa Fluor 488 was from Millipore. The CellTiter-Glo Luminescent Cell Viability Assay Kit was from Promega. The PE Annexin V Apoptosis Kit I was from BD Biosciences. Primary antibodies for Cdc42, Ras and Rab7 were from Santa Cruz Biotechnology, Thermo Fisher Scientific, and Sigma-Aldrich, respectively. 4-((*N*′-2-methylphenyl)ureido)-phenylacetyl-l-leucyl-l-aspartyl-l-valyl-l-prolyl-l-alanyl-l-alanyl-l-lysine (LDV) and its FITC-conjugated analogue (LDV-FITC) were synthesized by Commonwealth Biotechnology as described previously [30]. *N*-Formyl-Met-Leu-Phe-Phe (fMLFF) was purchased from Sigma-Aldrich. The CellLyticM Cell Lysis buffer and actin antibody were from Sigma-Aldrich. EGFR antibody was from Cell Signaling Technology. Goat anti-rabbit IgG with conjugated peroxidase and SuperSignal West Dura were both from Thermo Fisher Scientific. ChemiDoc XRS+ molecular imager and Image Lab software were from Bio-Rad Laboratories. The HyperCyt delivery system developed at the University of New Mexico is available from IntelliCyt and the CyAn ADP flow instrument was from Beckman Coulter. FACScan and Accuri C6 flow cytometer were both from BD Biosciences.

### Cell Culture

U937 FPRΔST cells (a gift from Dr. Eric Prossnitz, Dept. Cell Biology and Physiology, the University of New Mexico) were grown at 37°C in a humidified incubator with 5% CO_2_ and 95% air in RPMI 1640 supplemented with 2 mM L-glutamine, 100 units/mL penicillin, 100 μg/mL streptomycin, and 10% heat-inactivated fetal bovine serum. The cell line contains serine and threonine mutations in the *N*-formyl peptide receptor tail which prevent the phosphorylation mediated desensitization and extend the signal duration. SCC-12F cells (a gift from Dr. Laurie Hudson, Dept. Pharmaceutical Sciences, the University of New Mexico) were grown in a 1:1 combination of DMEM and nutrient mixture F-12 Ham’s medium supplemented with 10% fetal bovine serum. HeLa cells untransfected or stably overexpressing Rab7 wild-type (gifts from Dr. Matthew Seaman, Dept. Clinical Biochemistry, Cambridge Institute for Medical Research, Cambridge, UK) were grown in MEM with 10% serum. H358 cells (a gift from Dr. Kevan M. Shokat, the University of California, San Francisco) were grown in RPMI 1640 supplemented with 10% fetal bovine serum.

### Synthesis of Compound 1 and Its Analogues

The synthesis of CID1067700 (compound **1)** and some analogues has been reported previously [28]. Synthetic procedures for new analogues are described in the Supporting Information.

### Dose-Response Inhibition by Compound 1 and Its Analogues

The assay was carried out according to the protocols described previously [27, 31]. Briefly, individual GST-GTPases were separately attached to glutathione microsphere beads that were labeled with varying intensity of red fluorescent dye, and subsequently combined. The buffer contains 30 mM HEPES (pH 7.2), 150 mM KCl, 20 mM NaCl, 0.1% BSA, 1 mM DTT, 1 mM EDTA and 0.1% NP-40. For Rho A and Rac1, 1 mM EDTA was replaced with 1 mM MgCl_2_ to stabilize the protein. Compound **1** or its analogs at different concentrations was then added followed by BODIPY^-^FL GT(D)P. After incubation for 2 h at 4°C, the beads were delivered by HyperCyt for analysis on a CyAn ADP flow cytometer. The FL9 channel (ex/em: 635/750LP nm) was used to identify individual GTPase-coated bead sets while the FL1 channel (ex/em: 488/530 nm) measured the FITC fluorescence associated with the beads. To study whether the compounds are slow binders, BODIPY^-^ FL GTP was added either immediately or 2 h after compound addition. To study whether the compounds compete with BODIPY^-^ FL GTP, measurements of EC50 values were made at varying BODIPY^-^ FL GTP concentrations (25 nM, 100 nM, or 250 nM). As a control, compound **1** and its analogs were shown not to interfere with the fluorescence measurements of GST-GFP on glutathione beads. Therefore, the observed fluorescence decrease was attributed to *bona fide* inhibition of fluorescent guanine nucleotide binding and was not due to protein loss or fluorescence quenching. The curves were fitted to the sigmoidal dose-response equation using GraphPad Prism. Percent response was calculated as the ratio of (sample MCF–negative control MCF) / (positive control MCF–negative control MCF). For the positive control, DMSO instead of compounds was added; for the negative control, the compound was replaced with GTP at a concentration 5000 fold greater than [BODIPY^-^ FL GTP].

### Association and Dissociation Time Course

GTPases were bound to glutathione-coated microsphere beads overnight and unbound GTPases were removed by washing. The association and dissociation of BODIPY^-^ FL GTP was analyzed with the Accuri C6 flow cytometer in real-time. The initial 30 s set the baseline fluorescence. Then either BODIPY^-^ FL GTP at 100 nM by itself or with compound **1** at 50 μM was added. When the fluorescence plateau was reached, GTP at 50 μM was added to induce BODIPY^-^ FL GTP dissociation. In separate tests, when the binding of BODIPY^-^ FL GTP to GTPases stabilized, compound **1** at 50 μM was added. All the concentrations given are the final concentrations. To test the reversibility of compound **1**, GTPases were attached to the microsphere beads in the presence of 50 μM compound **1** overnight and beads underwent extensive washing to remove unbound GTPases or compounds. The binding of BODIPY^-^ FL GTP was compared to that without compound **1** pretreatment.

### GTPase and Effector Interaction Assay

The effect of compound **1** on the cellular activation status of Cdc42, Ras and Rab7, representing three generic subfamilies of the GTPases was measured using an effector interaction assay according to our previously established procedure [32]. GST effector chimeras consisting of the minimal GTPase binding domain were p21-activated kinase p21 binding domain (PAK-PBD) for Cdc42, Raf1 Ras-binding domain (Raf1-RBD) for Ras, and Rab-interacting lysosomal protein (RILP) for Rab7 binding measurements.

PAK-PBD and Raf1-RBD proteins were obtained from Millipore and RILP was purified according to established procedures [32]. Superdex peptide beads (13 μm, from GE Healthcare) derivatized with GSH were coated with effector proteins to saturation. 10,000 beads present an upper limit of 4 x 10^10^ sites or 3.3 nM in 20 μL. Incubating 800 nM (10 x Kd) GST effector protein with the GSH beads yields a bead site occupancy of ~91%. HeLa cells (10,000 cells/well in 12-well plate) were serum starved for 2 h and then treated with 10 μM compound **1** or 0.1% DMSO for 2 h. Next, cells were either left unstimulated or stimulated with 10 ng/mL epidermal growth factor (EGF, from Sigma-Aldrich) for 2 min. Cells were then lysed in 200 μL RIPA buffer (50 mM Tris-HCl, pH 7.4, 150 mM NaCl, 0.25% Na-deoxycholate, 1% NP40, 1 mM EDTA, 1 mM NaF, 1 mM PMSF, 1 mM Na_3_VO_4_, and protease inhibitors– 10 μg/mL each of chymostatin, leupeptin, pepstatin and antipain). The effector-coated glutathione beads (20,000 beads/assay) were incubated with 200 μL cell lysate from a single treated well for 1 h at 4°C. Bound and active GTPases were detected with a primary GTPase-specific antibody (given in Materials) and an Alexa 488 secondary antibody. The bead associated fluorescence representing bound GTPase was quantified on an Accuri C6 flow cytometer. Results are given as (sample MCF—negative control MCF)/positive control MCF where the negative control is the MCF reading obtained using lysates from unstimulated cells, and the positive control is the MCF reading obtained from the stimulated cells treated with DMSO.

### EGFR Degradation in SCC-12F Cells

SCC-12F cells were seeded in 12-well plates at 0.12 x 10^6^ cells/well and allowed to attach overnight. On the day of the experiment, the cells were starved in serum free medium containing 25 μg/mL cycloheximide for 2 h before compounds at different concentrations were added. The cells were treated with compound or DMSO for 0.5 h followed by the addition of ligand EGF at 20 nM. At time 0, 15, 30, 60, and 120 min, the suspension medium was removed and the cells were quickly washed with cold PBS and frozen at -80°C. For electrophoresis and blotting, CellLyticM Cell Lysis buffer containing protease inhibitor cocktails was added to the frozen cells and the protein concentrations were quantified using the Bradford assay. For SDS-PAGE, 10 μg of protein was loaded on each lane. After transblotting, the nitrocellulose membrane was probed with Anti-EGFR and Anti-actin primary antibody and secondary goat anti-rabbit IgG antibody with conjugated peroxidase. Actin served as a loading control. After adding the substrate SuperSignal West Dura, specific band signal was detected with ChemiDoc XRS+ molecular imager and the intensity was quantified using Image Lab software. Experiments were carried out either in duplicate and repeated or in triplicate. Results are given as the ratios of EGFR/actin at time t and EGFR/actin at time 0.

### EGFR Degradation in Rab7 Overexpressing HeLa Cells

The EGFR degradation assay in HeLa cells was carried out in a similar way to that in SCC-12F cells but with modifications. Briefly, HeLa cells overexpressing Rab7 wild type protein were serum starved overnight, incubated with 100 μM compound **1** for 3 h, and then treated with cycloheximide and stimulated with EGF in serum free medium for 0–180 min. Cell lysates were prepared at various time points and immunoblotted for total EGFR while actin served as a loading control. Experiments were carried out in triplicate.

### Integrin Activation Assay

The procedure followed the protocols described previously [30, 33, 34]. U937 FPRΔST cells (0.4~0.8 x 10^6^ cells/mL) were continuously stirred with a magnetic stir bar at 500 rpm in a test tube. The fluorescence was recorded on a FACScan flow cytometer. The baseline fluorescence of the cells was established for 30 s. Then LDV-FITC at 4 nM was added. After the fluorescence stabilized at ~120 s, the chemotactic ligand fMLFF at 100 nM was added for stimulation. When a plateau was reached at ~280 s, compounds at different concentrations were added to the cell suspension. All the concentrations given were the final concentrations. The percent inhibition was calculated according to Equation 1 (Supporting Information).

### Viability and Apoptosis Assay

The effects of compound **1** on the viability and apoptosis of H358 cells [35] and U937 FPRΔST cells were carried out in parallel for comparison. The CellTiter-Glo Luminescent Cell Viability Assay was used to measure the viability of H358 and U937 FPRΔST cells with or without compound **1** treatment. Cells were plated in a 96-well plate at 2000 cells per well and allowed to incubate overnight. Compound **1** or the same volume of DMSO was added on the second day. Viability was examined after 48 h. Data were collected from at least triplicate samples. Results were reported as viability treated/viability untreated (DMSO control). To evaluate apoptosis, the PE Annexin V Apoptosis Kit I was used. In 6-well plates, H358 cells were seeded at 50% confluence, while U937 FPRΔST cells were at 0.02 x 10^6^ /mL. After overnight incubation, the cells were treated with compound **1** or DMSO. At 24 h or 48 h, cells were washed with PBS, and resuspended in 100 uL annexin-V binding buffer. After staining with annexin-V-FITC and 7-AAD, cells were analyzed on an Accuri C6 flow cytometer. At least 7,000 cells were measured per individual sample. The annexin^+^AAD^-^ cells are defined as cells undergoing apoptosis. Data were collected from duplicate samples and the experiments were repeated. Results are given as the ratios of the apoptotic populations between the compound-treated and the DMSO-treated cells.

### Western Blotting to Detect Erk1/2 Phosphorylation Downstream of Ras Signaling

H358 lung cancer cells were seeded at 10^6^ cells/well in 6–well plates. For samples maintained in complete medium, cells were treated with 10 μM compound **1** or 0.1% DMSO for 1 h and lysed in 150 μl RIPA buffer (50 mM Tris-HCl, pH 7.4, 150 mM NaCl, 0.25% Na-deoxycholate, 1% NP-40, 1 mM EDTA, 1 mM NaF, 1 mM PMSF, 1 mM Na_3_VO_4_, protease inhibitors– 10 μg/ml each of chymostatin, leupeptin, pepstatin and antipain). For the stimulated samples, cells were serum starved for 2 h and treated with 10 μM compound **1** or 0.1% DMSO for 1 h. Then cells were stimulated with 10% FBS for 5 min and lysed in 150 μl RIPA buffer. Cell lysates were processed for SDS-PAGE, transferred to PVDF membranes and probed with a monoclonal antibody directed against phospho-Erk 1/2 (from Cell Signaling) and a monoclonal antibody directed against Erk 1/2 (from Cell Signaling) followed by an HRP labeled ECL anti-mouse IgG (from GE Healthcare).

### Statistical Analysis

Statistical analyses for individual assays included the Student t-test and One-way ANOVA and Dunnett’s post hoc multiple comparison test using GraphPad Prism version 5.0. In One-way ANOVA and Dunnett’s post hoc multiple comparison test, all compound **1** or analogue treatment groups were compared with the DMSO treatment control group. Statistical analysis of compound effects toward Erk signaling was conducted with an unpaired two-tailed t-test using GraphPad Prism. All values of *p* < 0.02 were defined as significant and highlighted with *.

## Results

### Compound 1 Inhibits GTP Binding to GTPases

**Fig 1:**
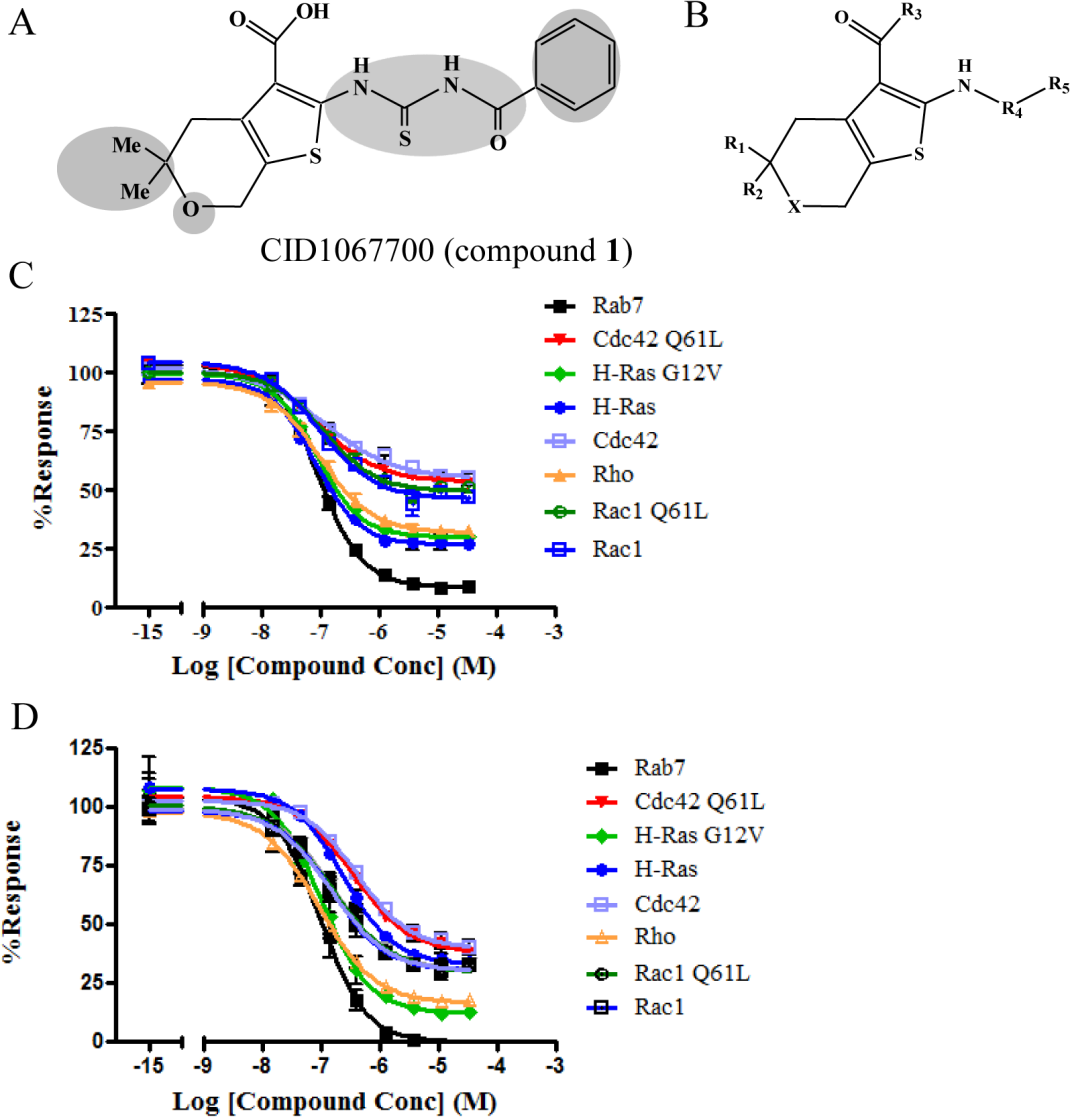
Compound 1 structure and dose-response curves. (A) Structure of **1** and sites of functionalization. Shaded areas are modified regions for SAR. (B) General structure of compound **1** and analogues. (C) The concentration dependence of compound **1** on BODIPY^-^ FL GTP binding to eight GTPases. (D) The concentration dependence of compound **1** on BODIPY^-^FL GDP binding to eight GTPases. Data shown in (C) are representative of at least three independent sets of measurements with each set conducted in duplicate. All the data points from each set were used to fit the sigmoidal dose-response equation. Percent response was calculated as the ratio of (sample MCF–negative control MCF) / (positive control MCF–negative control MCF). For the positive control, DMSO instead of the compound was added; for the negative control, the compound was replaced with GTP at a concentration 5000 fold greater than BODIPY^-^FL GTP. MCF stands for median channel fluorescence.

Our previous studies have shown that the GST-tagged GTPases used in our assays are only minimally loaded with guanine nucleotide and can be charged with fluorescently labeled GTP [19]. The effect of compound **1** (Fig 1A) on the equilibrium binding of BODIPY^-^ FL GTP to eight different GTPases was comparatively assessed in a multiplex assay. The eight GTPases were: H-Ras and its G12V constitutively active mutant, Cdc42 and its Q61L constitutively active mutant, Rac1 and its Q61L constitutively active mutant, RhoA and Rab7. These are representative members of Ras, Rho and Rab GTPases. The compound inhibited BODIPY^-^ FL GTP binding to all the GTPases tested in a dose-dependent manner (Fig 1C, Table 1). The fluorescence decrease was confirmed to be due to *bona fide* inhibition of BODIPY^-^ FL GTP binding and not a result of protein loss or fluorescence quenching. The nucleotide identity appears not to be essential since compound **1** also inhibited BODIPY^-^ FL GDP binding (Fig 1D, Table 1). The concentration of the fluorescent guanine nucleotide in the assay was close to its Kd. According to the Cheng-Prusoff equation, the EC50 obtained is therefore an estimate of the Ki of the compound [36]. Remaining activities were observed with different GTPases at varying levels even at high compound concentrations. In the many repetitions that were carried out, some GTPases always have higher remaining activity than others. For example, Ras always has higher remaining activity than Rab7, even when different batches were tested. In a single set of experiment, proteins from different sources showed almost identical dose-response curves (Table 2). Therefore it suggests that the observed difference among different GTPases is not due to any enzyme preparation differences but instead is likely caused by inherent properties of individual GTPases. Moreover, protein denaturation is not likely to be the main contributor of the observed remaining fluorescence at high compound concentrations either (as discussed in the Supporting Information). Instead, the remaining fluorescence may suggest incomplete blocking by the compound. The pan-GTPase inhibitory activity is likely attributed to the targeted binding towards the common nucleotide binding site, as previously documented for Rab7 and supported by *in silico* docking studies[28]. However, due to the sequence and structure variations among GTPases, the compound may fit differently into individual binding pockets as reflected by the difference in both the affinity and the remaining activity. The binding difference might be a starting point for selective inhibitor development. Structural information will greatly facilitate the process and is being actively pursued.

**Table 1:** EC50s of compound 1 and its analogues.

compound #	CID	structure derivation	Rab7	H-Ras	H-Ras G12V	Cdc42	Cdc42 Q61L	RhoA	Rac1	Rac1 Q61L
1	1067700	X = O;R_1_ = Me;R_2_ = Me;R_3_ = OH;R_4_ = CS-NH-CO; R_5_ = phenyl	0.12 ± 0.055	0.16 ± 0.16	0.052 ± 0.044	1.09 ± 1.62	0.047 ± 0.038	0.062 ± 0.049	0.062± 0.049	0.075 ± 0.059
1^a^	1067700	X = O;R_1_ = Me;R_2_ = Me;R_3_ = OH;R_4_ = CS-NH-CO; R_5_ = phenyl	0.43	0.24	0.26	0.42	0.35	0.10	0.099	0.12
**2**	1251121	R_3_ = OMe	>1000	>1000	>1000	>1000	>1000	NA	NA	NA
**3**	46916266	R_1_ = H;R_2_ = H	0.16 ± 0.067	0.17 ± 0.094	0.12 ± 0.12	0.11 ± 0.064	0.13 ± 0.078	0.23 ± 0.21	0.18 ± 0.13	0.23 ± 0.22
**4**	740871	R_4_ = CO-CH2	>1000	>1000	>1000	>1000	>1000	NA	NA	NA
**5**	1280844	R_4_ = CS-NH	NA	NA	3.55	391.00	125.70	1.39	4.06	1.66
**6**	46916265	R_4_ = CO-NH-CO	0.31 ± 0.09	0.44 ± 0.41	0.49 ± 0.48	17.00	0.47	0.33 ± 0.03	0.35 ± 0.05	0.40 ± 0.02
**7**	53301934	R_5_ = 3-MeO-phenyl	0.15 ± 0.13	0.13 ± 0.13	0.12 ± 0.12	0.58 ± 0.83	0.50 ± 0.71	0.20 ± 0.13	0.19 ± 0.06	0.22 ± 0.14
**8**	53301932	R_5_ = 4-MeO-phenyl	0.14 ± 0.07	0.14 ± 0.09	0.17 ± 0.10	1.2 ± 1.0	0.23 ± 0.039	0.15 ± 0.06	0.13 ± 0.06	0.15 ± 0.09
**9**	53377405	R_5_ = 4-F-phenyl	0.41 ± 0.19	0.33 ± 0.22	0.091	1.0± 1.2	0.88 ± 0.47	0.11	0.15	0.13
**10**	53301931	R_5_ = 2-Br-phenyl	0.43 ± 0.68	0.62 ± 1.00	0.43 ± 0.68	46.77 ± 66.01	14.76 ± 20.80	0.48 ± 0.74	0.60 ± 0.80	0.42 ± 0.65
**11**	53377404	R_5_ = 2-Me-phenyl	0.41	0.44	0.38	2.66	1.35	0.49	0.44	0.37
**12**	53301933	R_5_ = 4-Br-phenyl	0.30 ± 0.18	0.33 ± 0.24	0.32 ± 0.22	25.86 ± 44.07	10.89 ± 18.37	0.32 ± 0.13	0.26 ± 0.11	0.28 ± 0.15

**Fig 2:**
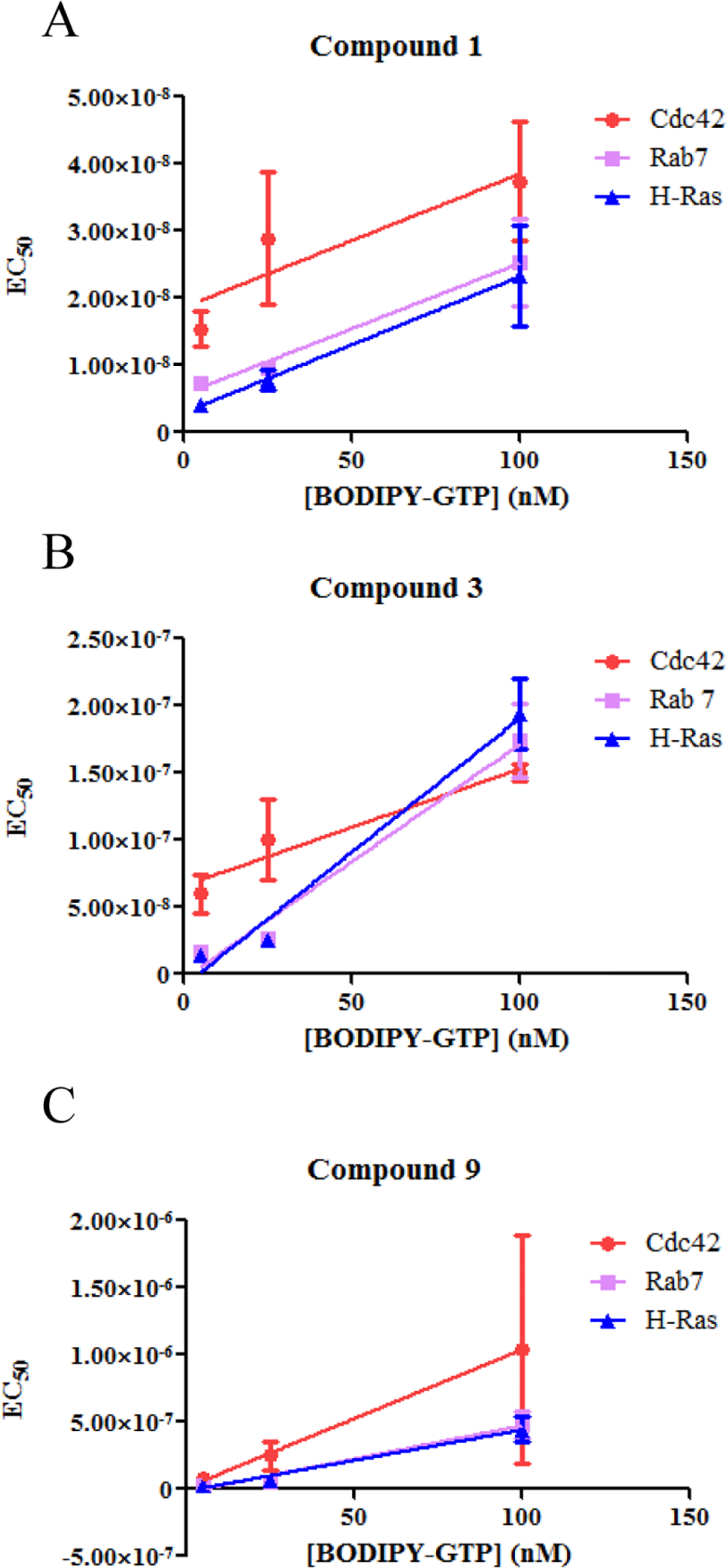
Correlation between EC50 values and the concentration of BODIPY^-^FL GTP. The EC50 values of compound **1** and its analogues increased almost linearly (*R* ^2^ > 0.7) when the concentration of BODIPY^-^FL GTP increased. A: compound **1**; B: compound **3**; C: compound **9**. The linear changes suggest a competitive inhibition mechanism [37]. The experiments were carried out in triplicate.

Derivatization sites were indicated in Fig 1. Compound numbers are used in the main text for convenience. For EC50s with standard deviations, the values were the averages from multiple measurements. In multiple repetitions, variability was observed with Cdc42, which increased its EC50 average. Therefore, the biochemical data for Cdc42 when needed for comparison is from a single set of experiment (Table 2 and Fig 2). For EC50s without standard deviations, the values were determined from one measurement carried out in duplicate where all the data points were used to fit the sigmoidal dose-response equation. EC50 values were noted NA when they were not available or data could not give acceptable fit.

^*a*^The concentration dependence on BODIPY^-^FL GDP.

**Table 2:** Preincubation of compound 1 with GTPases had no effects on EC50s.EC50

	Cdc42_1	Rab7_1	Ras	Rab7_2	Cdc42_2
without incubation	2.23 ± 1.47	0.14 ± 0.0019	0.13 ± 0.0026	0.16 ± 0.0011	4.46 ± 2.63
with incubation	2.06 ± 0.038	0.11 ± 0.0027	0.10 ± 0.0035	0.12 ± 0.0023	3.71 ± 0.039

The incubation time was 2 h before the addition of BODIPY^-^FL GTP. The experiments with and without incubation were carried out in parallel on the same day, in duplicates and repeated. Cdc42_1 was purified in house while Cdc42_2 was purchased from Cytoskeleton. Rab7_1 and Rab7_2 were from two separate preparations purified in house.

### Compound 1 Is a Reversible and Mostly Competitive Inhibitor

Initial studies showed that compound **1** is a competitive guanine nucleotide binding inhibitor of Rab7 [28]. The mechanisms of inhibition by the compound towards several GTPases were further examined here either by comparing EC50 values obtained under different conditions or through kinetic binding and dissociation studies. To test whether compound **1** is a slow binder, the GTPases were treated with compound **1** followed by either a two-hour incubation or no incubation before BODIPY^-^ FL GTP was added. The EC50 values thus obtained were almost identical (Table 2, *p* > 0.5), suggesting that the compound is not a slow binder. Slow-binding can be characteristic of non-competitive inhibition. For competitive inhibitors, the EC50 values increase linearly when the concentration of the ligand BODIPY^-^ FL GTP increases [37]. When different concentrations of BODIPY^-^ FL GTP were used, the EC50 values of compound **1** and its analogs increased almost linearly along with the concentration increase of BODIPY^-^ FL GTP suggesting that compound **1** and its analogs are largely competitive inhibitors (Fig 2). Deviations from straight lines were observed in some cases implying non-classical competitive behaviors (Fig 2A, Cdc42).

**Fig 3:**
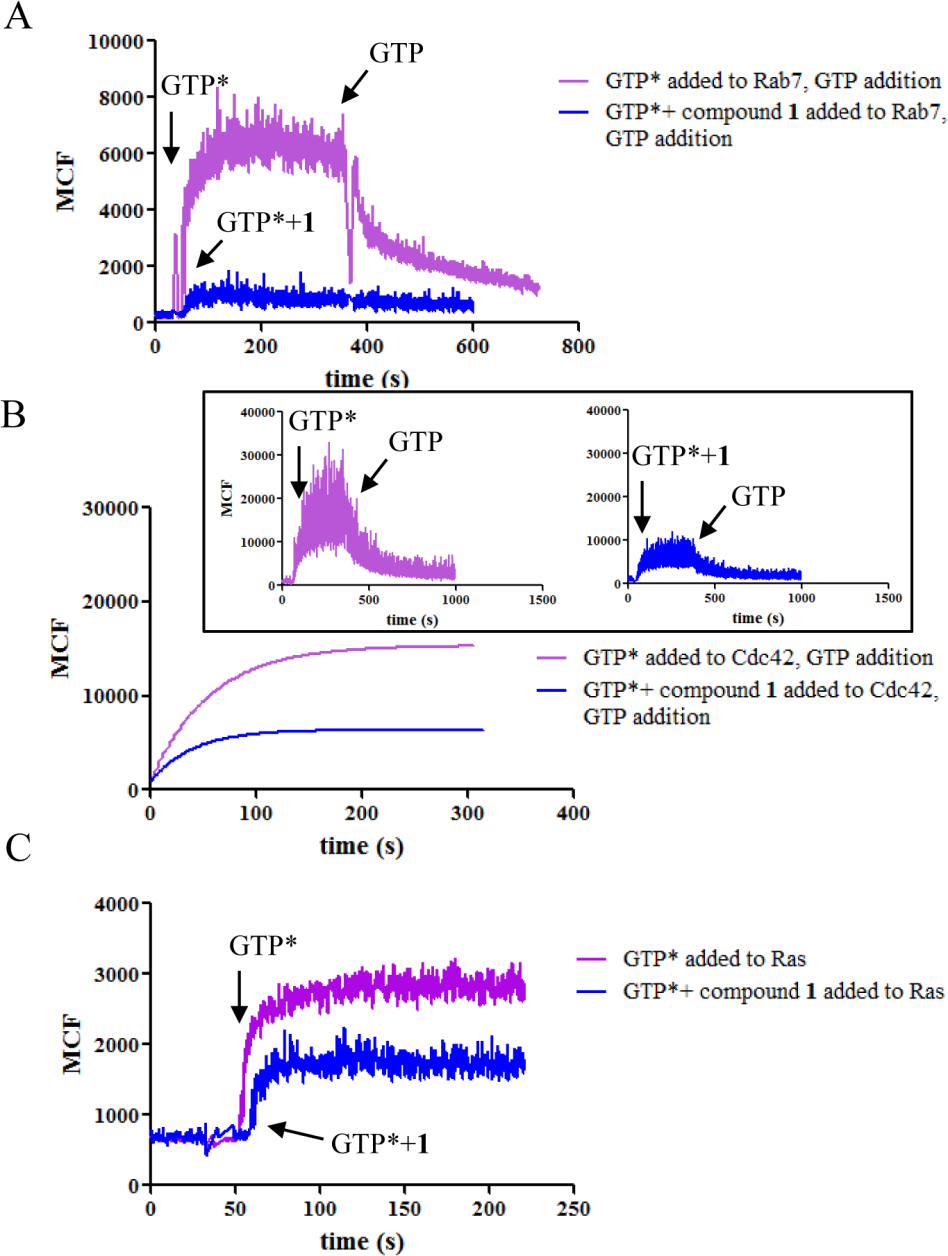
Compound 1 inhibits BODIPY^-^FL GTP binding to GTPases. Towards Rab7 (A), Cdc42 (B) and Ras (C) in kinetic assays. BODIPY^-^FL GTP was added in the presence or absence of compound **1**. The experiments were conducted in triplicate and representative curves were shown. For Cdc42, the fluorescence was fitted to a single exponential equation due to noisy signals. Shown in the inset are the original data. GTP* represents BODIPY^-^FL GTP.

**Fig 4:**
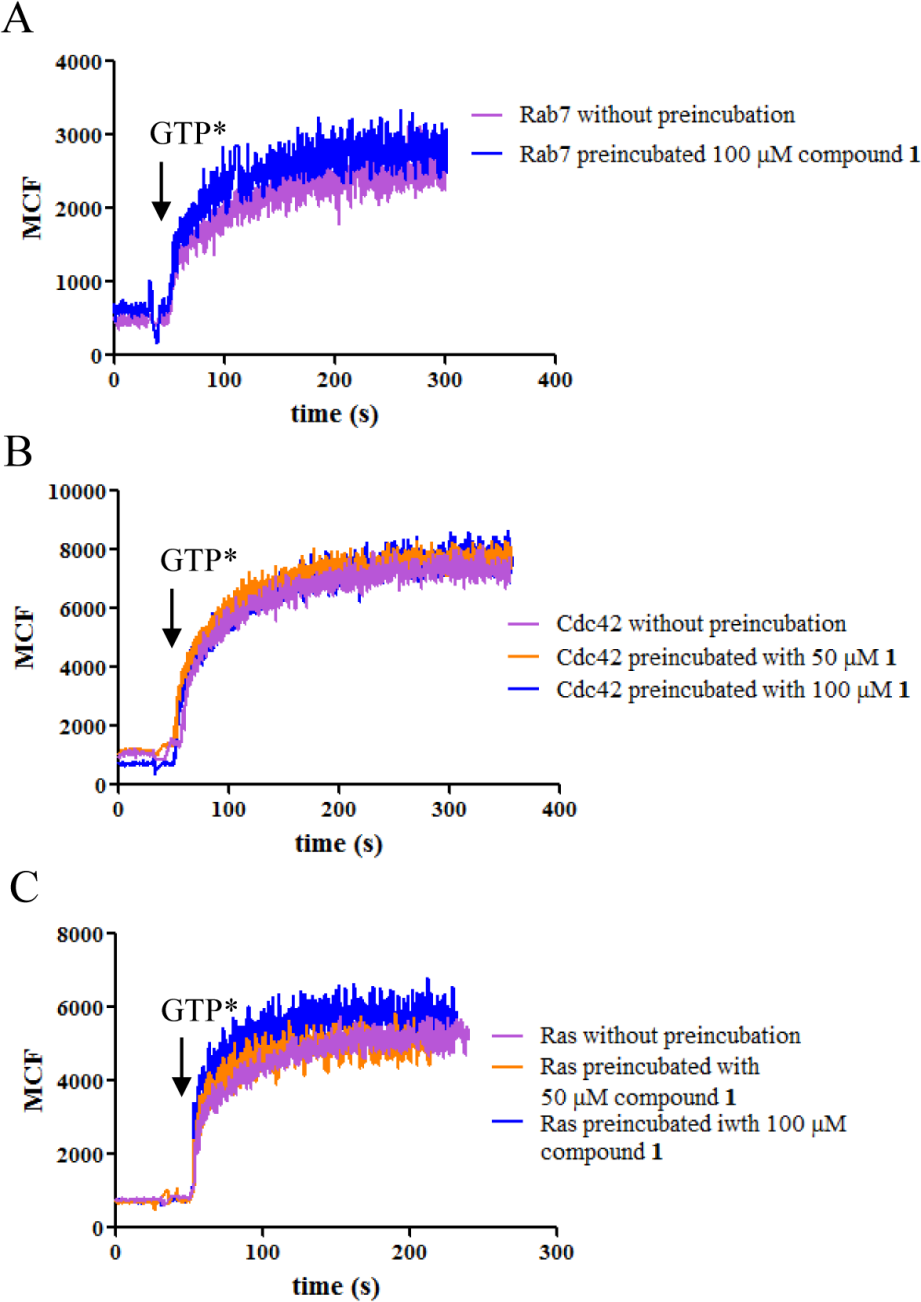
Lack of covalent bond formation between GTPases and compound 1. Rab7 (A), Cdc42 (B) or Ras (C) was incubated with or without compound **1** overnight followed by extensive washing. Binding of BODIPY^-^FL GTP to GTPases was indistinguishable whether the enzymes had been treated with compound **1** or not.

**Fig 5:**
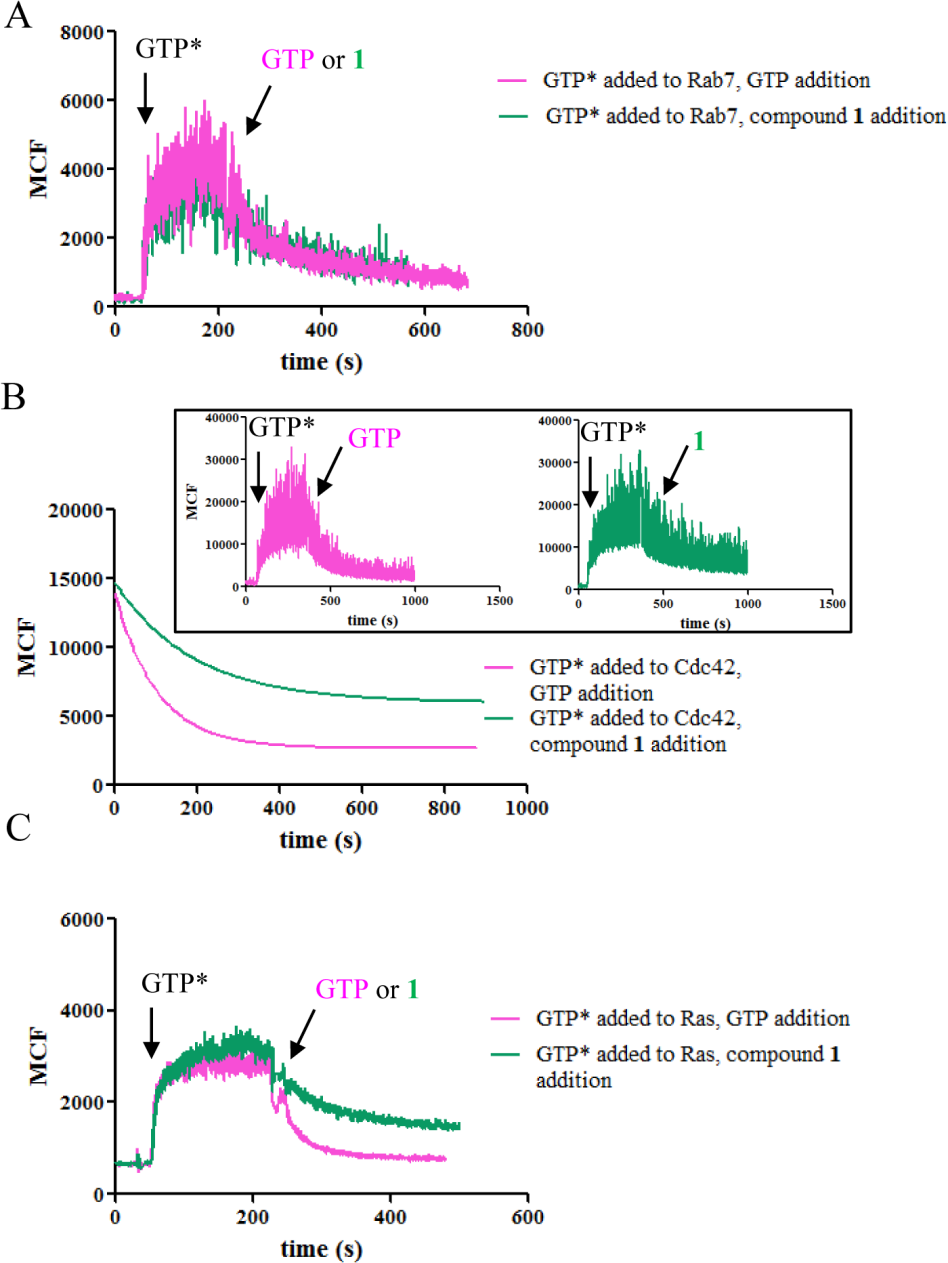
Compound 1 and GTP induced BODIPY^-^FL GTP dissociation. From Rab7 (A), Cdc42 (B) and Ras (C) in kinetic assays. After the binding of BODIPY^-^FL GTP to the GTPases reached plateau, either compound **1** or GTP at the same concentration, was added. The experiments were conducted in at least triplicate. For Cdc42, the fitted data were shown while the inset showed the original data.

In kinetic studies, the binding of BODIPY^-^ FL GTP was decreased for several GTPases when tested in the presence of compound **1** showing the inhibitory activity of the compound towards guanine nucleotide binding (Fig 3A–3C). Whether or not the GTPases were pretreated with compound **1**, the proteins showed comparable ability to bind BODIPY^-^FL GTP after unbound compound was removed by extensive washing (Fig 4A–4C). Therefore, compound **1** did not form a covalent bond with GTPases and the inhibition was reversed when the compound was removed. When GTPases were first loaded with BODIPY^-^FL GTP, either non-fluorescent GTP or compound **1** at the same concentration were able to induce dissociation of BODIPY^-^FL GTP (Fig 5A–5C). Residual fluorescence was observed for Ras and Cdc42, which is reminiscent of the remaining activity noted in the equilibrium dose response curves. Combined with previous studies [28], the results suggest that compound **1** is mostly a competitive inhibitor. It shows more classical competition mechanism towards Rab7 as indicated by nearly complete inhibition at high compound concentration, fast and reversible binding, and linear variation between EC50 and [BODIPY^-^ FL GTP], while for other GTPases, deviation from the classical mechanism was observed as shown by the incomplete inhibition and nonlinear relation between EC50 and [BODIPY^-^FL GTP]. The discrepancy is possibly due to the structural differences at the nucleotide binding sites among different GTPases where the compound fits differently.

### SAR of Compound 1 Shows Essential Functional Groups

The scaffold was structurally modified to identify the components important for the inhibitory activity. Modifications were focused on four regions of the scaffold (shaded regions of Fig 1A, generalized structure 1B, and Table 1). The carboxylic acid moiety was shown to be essential. Esterification of this functionality (compound **2**) resulted in loss of activity for all GTPases tested. The pyran ring was tolerant to alkyl substitution, as replacement of the gem-dimethyl moiety with a simplified methylene group (compound **3**) did not cause a significant change in potency. Replacement of the sulfur atom with an oxygen atom (compound **6**) led to more than 2-fold loss in potency across all the GTPases. Replacing the thiocarbonyl-NH-carbonyl with the carbonyl-CH_2_ unit (compound **4**) or removing the carbonyl (compound **5**) and thus shortening the linker led to inactive analogs for all GTPases. Substitutions on the phenyl ring (compound **7**–**12**) did not provide clear SAR indications except that bromine decreased the inhibitory potency against Cdc42 whether it was at *ortho*- or *para-* position. The complete data on all the tested analogues are summarized in S1 Table.

### Compound 1 Inhibits the Interactions between GTPases and Their Effectors

**Fig 6:**
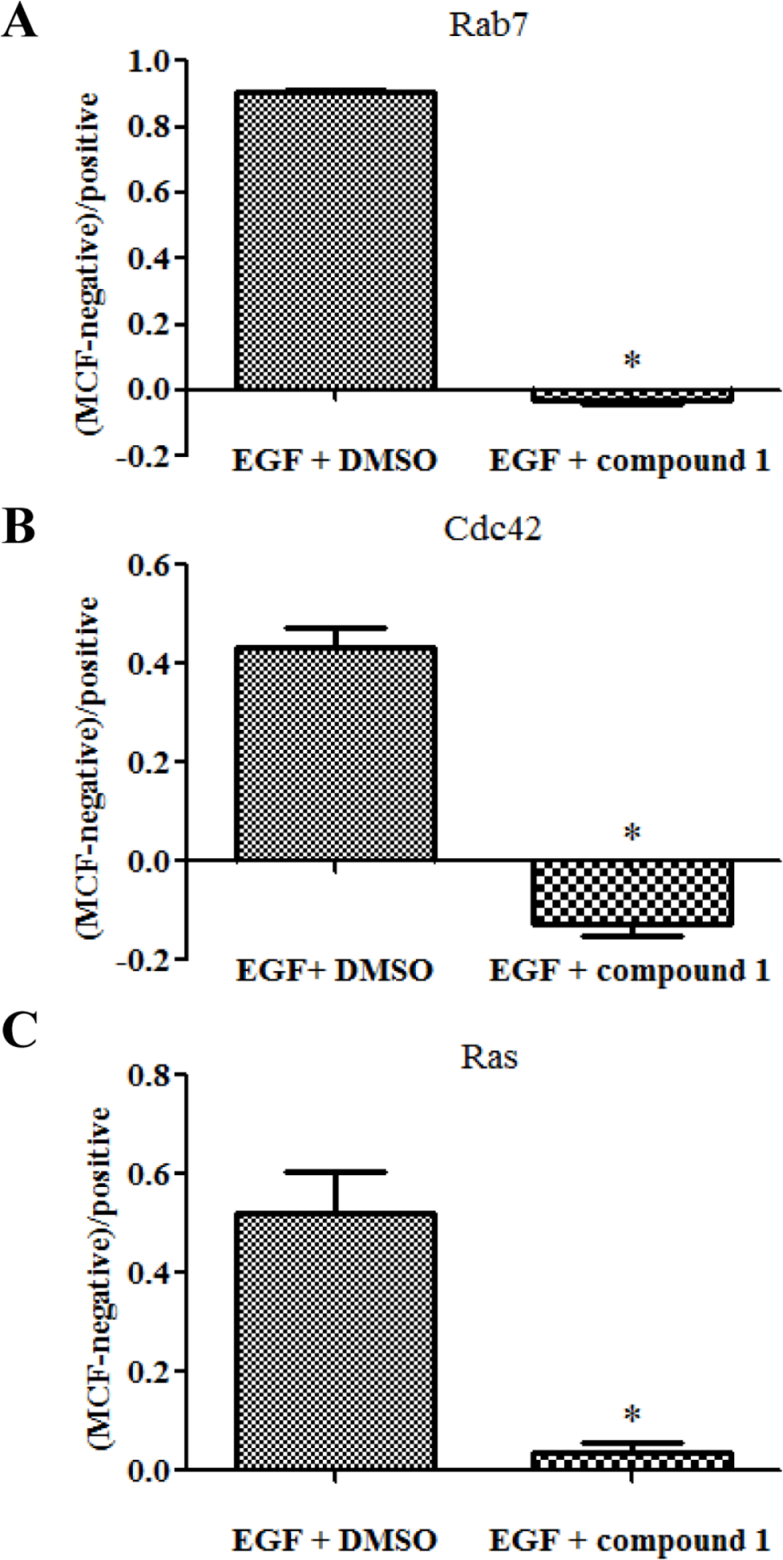
Compound 1 inhibited cellular activation of GTPases. Rab7 (A), Cdc42 (B), and Ras (C) in response to growth factor stimulation. HeLa cells were treated with sequential starvation, compound addition and EGF stimulation. Active GTPases in the cell lysates were quantified using effector linked glutathione beads, probed with fluorescent antibody and analyzed on flow cytometer. Results are given as (sample MCF—negative control MCF)/positive control MCF where the negative control is the MCF reading obtained using lysates from unstimulated cells, and the positive control is the MCF reading obtained from the stimulated cells treated with DMSO.

Compound **1** blocks guanine nucleotide binding to GTPase by competing for the nucleotide binding site. It is thus interesting to know whether the compound maintains the GTPase in an active state mimicking the GTP bound conformation or induces an inactive conformation in cellular environments. The effects of compound **1** on the cellular GTPase activation status was examined using a quantitative effector binding assay [32]. After a short period of growth factor stimulation, robust levels of active and GTP-bound Cdc42, Ras and Rab7 can be detected (Fig 6, EGF+DMSO). However, the activation was dramatically blunted by pretreatment with compound **1** prior to growth factor addition (Fig 6, EGF+compound **1**). The basal level of active GTPase in cells (unstimulated,-EGF) was normalized to zero. For Cdc42 and Rab7, compound **1** decreased the levels of active GTPases in cells below the basal levels. Therefore, when compound **1** binds to the nucleotide binding site, it induces or maintains an inactive GTPase conformation that is unable to bind downstream effectors.

### Compound 1 Inhibits EGFR Receptor Degradation Regulated by Rab GTPases

**Fig 7:**
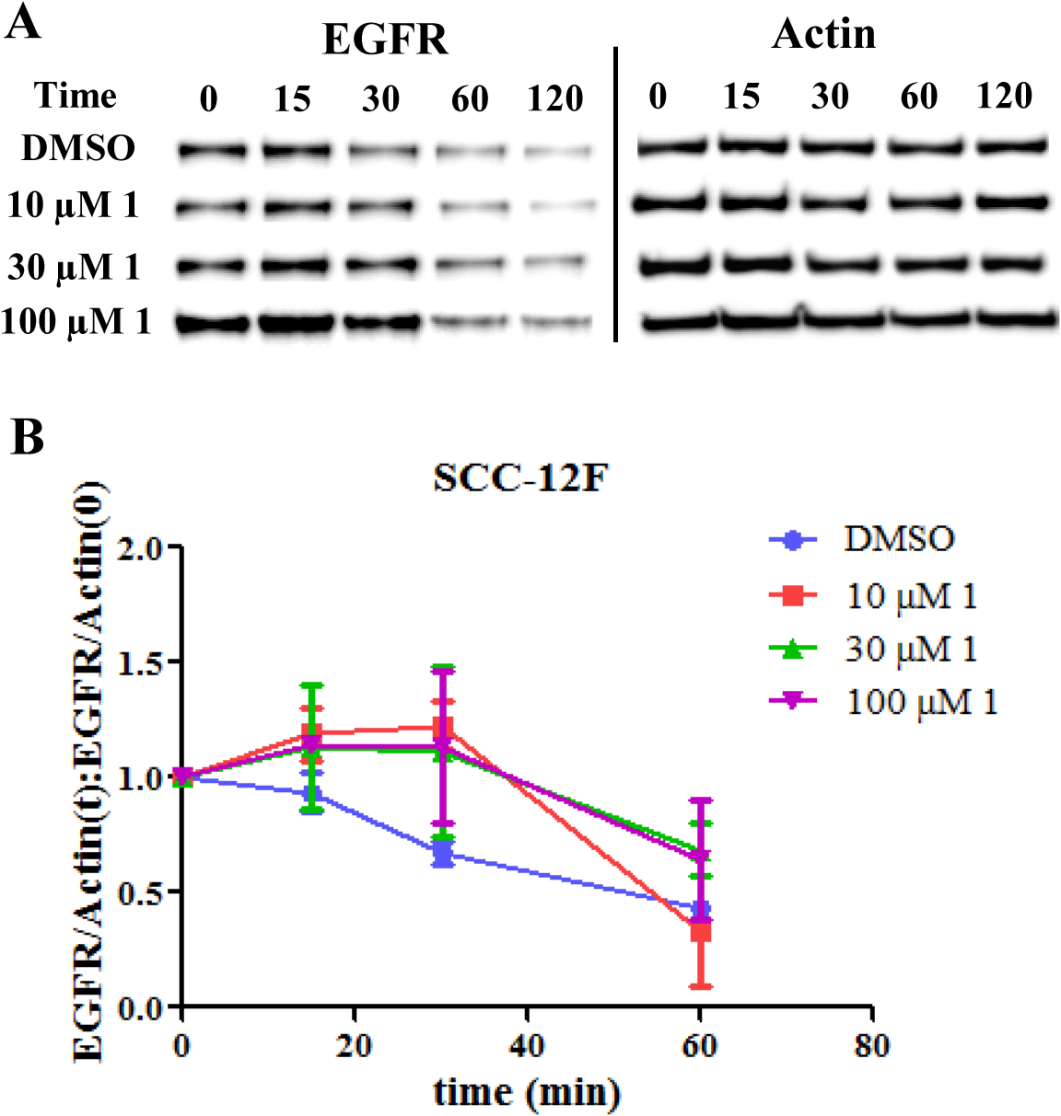
Compound 1 inhibited EGFR degradation in SCC-12F cells. A. Immunoblots of EGFR at different time points after treatment with **1** at different concentrations or DMSO. Actin served as a loading control. Each experiment included duplicate samples and was repeated in triplicate. B. Time course of EGFR degradation. The ratio of EGFR to actin was quantified at different time points and compared to time zero when ligand EGF was added.

**Fig 8:**
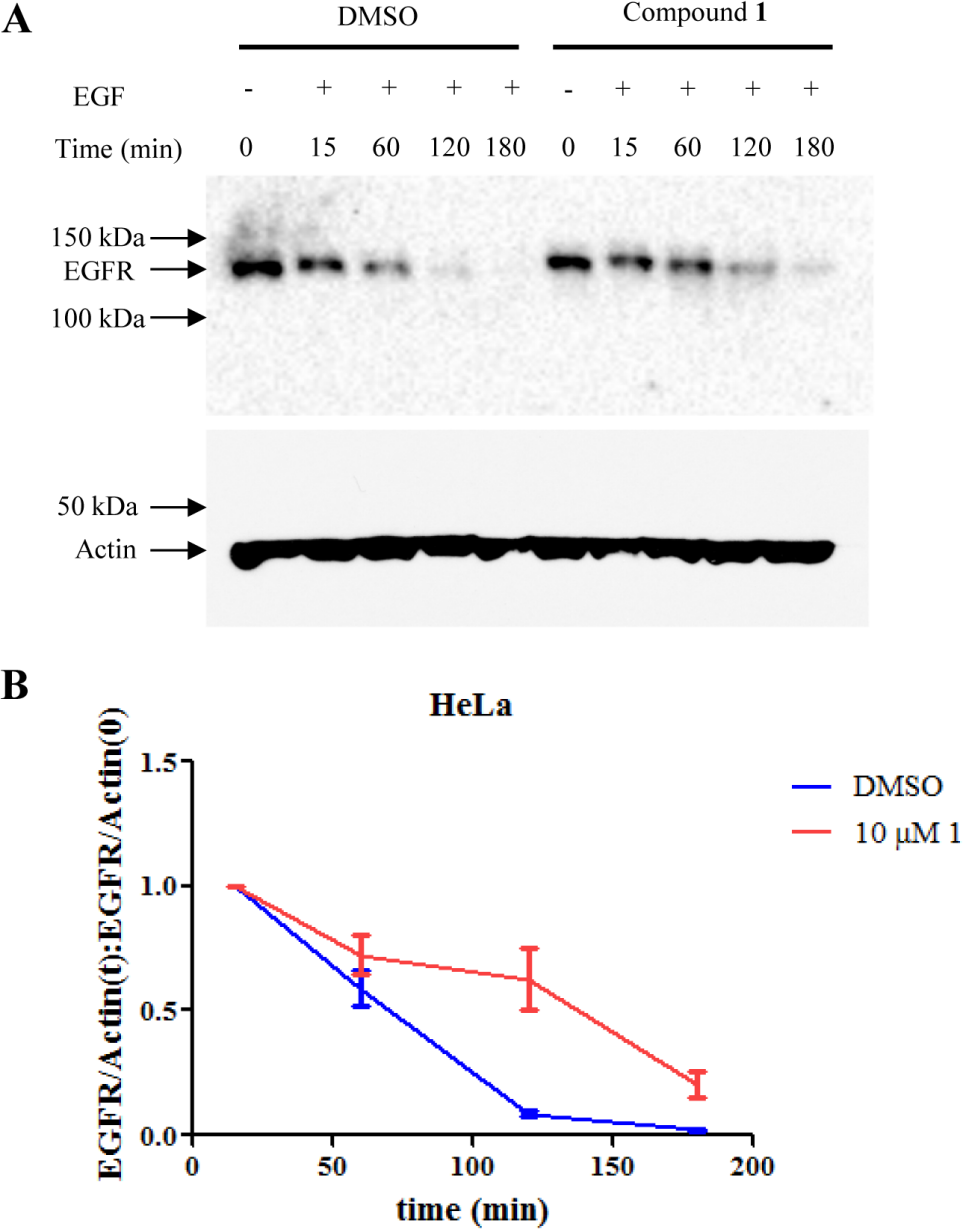
Compound 1 inhibited EGFR degradation in Rab7-expressing HeLa cells. A. Immunoblots of EGFR at different time points after treatment with **1** or DMSO. B. Time course of EGFR degradation. Data were processed as in Fig 7. The experiments were replicated three times.

The capacity of compound **1** to functionally inhibit Rab GTPases was examined using an EGFR degradation assay. It is well established that Rab subfamily GTPases regulate the trafficking and degradation of macromolecular cargos, such as the tyrosine kinase epidermal growth factor receptor, EGFR [4, 38]. After being activated by the EGF ligand, receptor internalization through endocytosis is stimulated. EGFR is transferred first to the early endosome, then to the late endosome and finally degraded in the lysosome which terminates ligand induced signaling. Rab5 regulates delivery to the early endosome, while receptor delivery to the late endosome and lysosome is controlled by Rab7. The effect of compound **1** on EGFR degradation kinetics was studied to assess the functional inhibition of Rab-regulated pathways. Two cell lines, SCC-12F with well-described EGFR degradation kinetics [39] and HeLa cells overexpressing wild type Rab7 were tested in this assay. Cycloheximide was included as a protein synthesis inhibitor to prevent *de novo* synthesis of the receptor. Compared with DMSO-treated control cells, treatment with compound **1** delayed EGFR degradation in both cell lines (Figs 7 and 8). EGFR degradation in HeLa cells was delayed at later time points compared with SCC-12F cells which may reflect the overexpression of wild type Rab7. A slowdown of EGFR degradation was similarly observed when Rab5 and Rab7 were inactivated by mutation or deletion [38, 40]. Analogs of compound **1** also delayed receptor degradation when tested (S1 Fig). Together, the data indicate that compound **1** and its analogues can inhibit Rab GTPases in cells.

### Compound 1 Inhibits Rho GTPases-involved Integrin Activation

**Fig 9:**
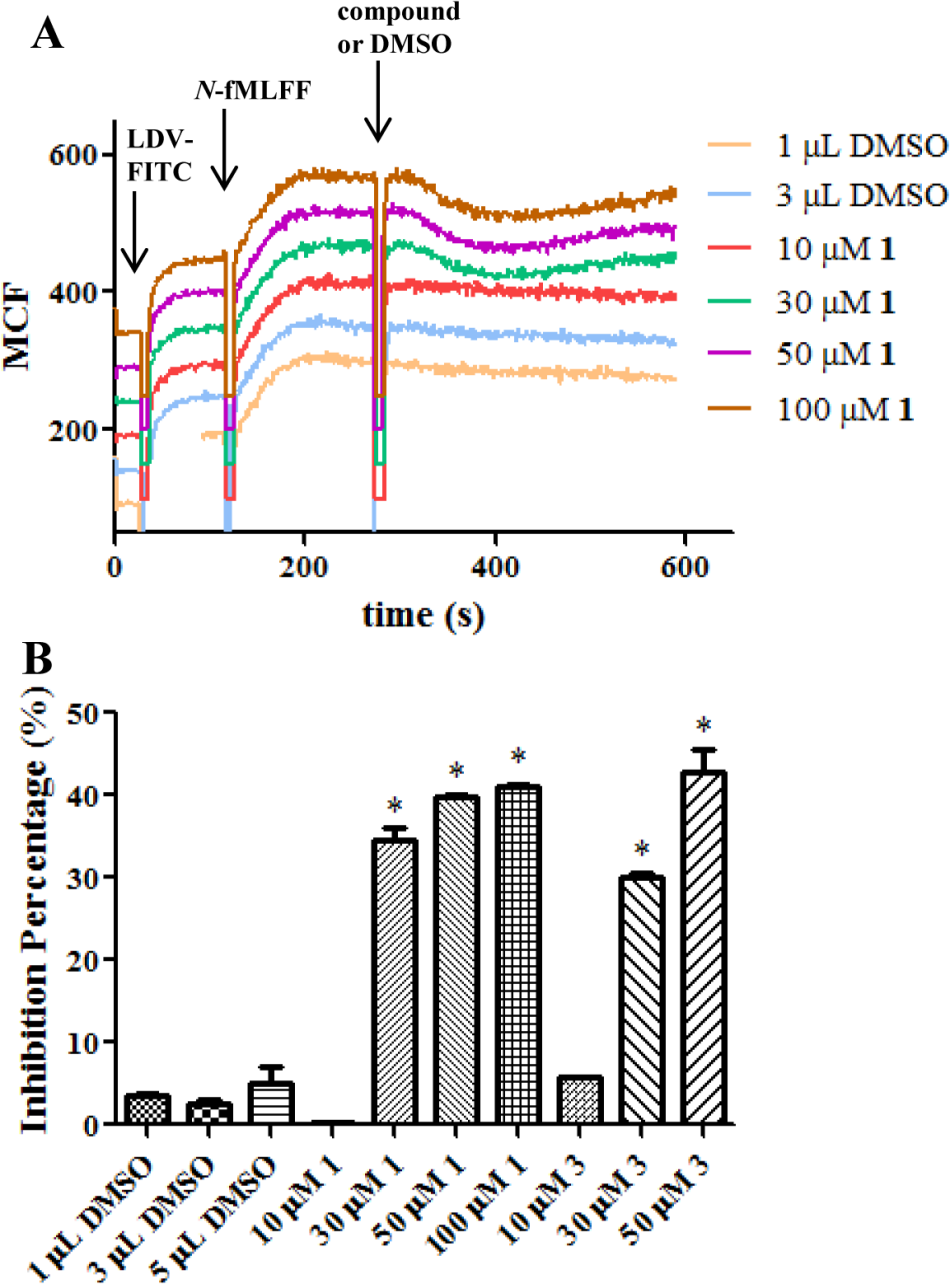
Compound 1 inhibited the integrin VLA-4 activation. **A.** LDV-FITC binds to VLA-4 in the resting state, while the *N*-fMLFF addition led to high-affinity binding that was inhibited by **1**. Note that the different curves of fluorescence reading versus time are graphed in a staggered manner for ease of viewing. B. Percent Inhibition for compounds **1** and **3**. The percent inhibition was calculated according to Equation 1 in Supporting Information.

Whether compound **1** could inhibit Rho GTPases at the functional level was examined in an integrin activation assay. At the site of injury, chemotactic agents released by infectious agents stimulate integrin Very Late Antigen-4 (VLA-4) on the leucocyte to assume a high affinity conformation which can bind to the CS-1 region of fibronectin in endothelial cells. The arrest of the leucocyte will then initiate a cascade of immune responses [41]. Rho GTPases are known to play important roles in transducing the signals from the chemotactic agent to the integrin [42, 43]. The integrin affinity change was monitored in real-time by an established assay that utilizes a fluorescein isothiocyanate labeled LDV peptide, LDV-FITC [30]. LDV contains the fragment Leu-Asp-Val of fibronectin that binds to VLA-4 expressed on the U937 FPRΔST cells. The initial binding of LDV-FITC represents the affinity of VLA-4 in the basal state (Fig 9A). Stimulation with the formyl-MLFF peptide, a mimic of the chemotactic agent, induced a VLA-4 affinity increase resulting in increased LDV-FITC binding. A Cdc42 selective inhibitor, CID2950007, has previously been shown to decrease the integrin activation in a dose-dependent fashion [19]. The addition of compound **1** or its analogs blocked the Rho GTPases involved signal transduction and caused diminished binding, while the addition of the same volume of DMSO had no effect. The loss of binding caused by the compounds suggested a reduced affinity between LDV-FITC and the cells. This was corroborated by an observed increase of LDV-FITC dissociation induced by a large excess of non-fluorescent LDV (S2 Fig). The blockade was temporary and the fluorescence level later recovered. Considering the dynamic nature of the signal transduction, it is possible that redundant pathways can serve to overcome the GTPase inhibition with time. Analogue compound **3** also dose-dependently blocked integrin activation (Fig 9B). It is nonetheless noted that the correlation between EC50 values and the phenotypic activity to block integrin activation is not straightforward since several members in the Rho subfamily GTPases are involved.

### Compound 1 Affects Ras-dependent Viability, Apoptosis and Erk Signaling

**Fig 10:**
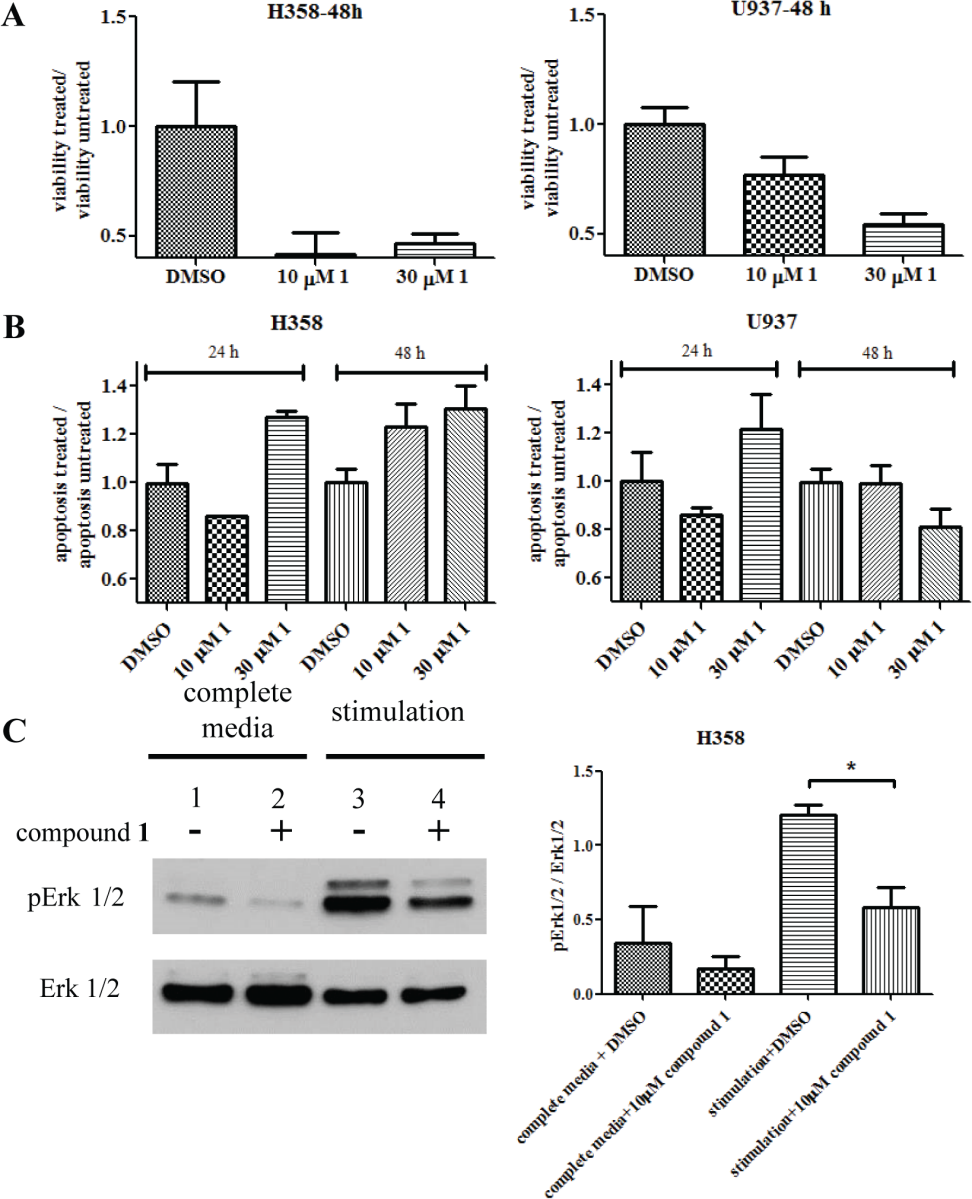
Effects of compound 1 on the Ras-dependent H358 cells. The U937 FPRΔST cell line was used as control since it had no known Ras-dependency. (A) Compound **1** decreased the viability of H358 cells at 48 h, compared to the control. (B) Apoptosis analysis showed increase response of H358 cells over time compared to control cells. (C) Treatment of compound **1** decreased the phosphorylation level of ERK 1/2 in H358 cells. (-) 0.1% DMSO treated controls; (+) 10 μM compound **1** treated samples. Cells were either grown in complete medium (lane 1 and 2) or stimulated after starvation (lane 3 and 4). The experiment was conducted three times. A representative immunoblot is shown. Immunoblots from all three experiments were quantified by densitometry using exposures in the linear range. The relative ratios of phospho Erk1/2 to total Erk1/2 are plotted. *p* = 0.0142, calculated with an unpaired two-tailed t-test using GraphPad Prism.

To measure the inhibitory activity of compound **1** towards Ras function in a cellular environment, its effects on the viability and apoptosis of Ras-dependent cells were tested. The working hypothesis is that if compound **1** inhibits intracellular Ras, then treatment with the compound will decrease the viability and increase the apoptosis of a Ras-dependent cell line while having lesser effects on Ras-independent cells. H358 cells were chosen for their established dependence on Ras GTPase [35]. Another cell line U937 FPRΔST with unknown Ras dependency was used as a control. We observed that compound **1** decreased the viability of H358 cells more than the U937 FPRΔST cells (Fig 10A). Apoptosis in both cell lines was also explored. A time-dependent apoptosis increase was observed in H358 cells, which was absent in U937 FPRΔST cells (Fig 10B). As an independent measure of the compound **1** effect on Ras signaling, the phosphorylation status of Erk 1/2 downstream of the Ras-activated survival pathway was examined by immunoblotting. Cells were either maintained in complete medium or stimulated with FBS after starvation (Materials and Methods). In both cases, the phosphorylation level of Erk 1/2 was decreased by compound **1** treatment (Fig 10C).

## Discussion

Our data shows that compound **1** can inhibit guanine nucleotide binding to multiple GTPases. The mechanism of inhibition of the compound towards Rab7 has been studied before, and it was demonstrated that compound **1** directly competes with GTP or GDP for the guanine nucleotide binding site which was supported by molecular docking analysis [28]. However, mechanistic studies towards other GTPases and more detailed characterization have not yet been reported. Our results show that as a pan-GTPase inhibitor, compound **1** is largely a reversible competitive inhibitor, and binds to the nucleotide binding site on GTPases. This is supported by several observations: the compound blocked guanine nucleotide binding to the GTPases in both equilibrium and kinetic assays (Figs 1 and 3); there was no slow binding behavior (Table 2); no covalent bonds were formed between the compound and the GTPases (Fig 4); the EC50 values of the compound and its analogues varied mostly linearly with the concentration of BODIPY^-^FL GTP (Fig 2). On the other hand, considering the varying effects that compound **1** has on different GTPases, it is possible that the compound could fit into the site of Rab7 in a more classical competitive manner but for the other GTPases, the fit is less complementary as reflected by the observed remaining activity (Figs 1C, 1D and 5) and the divergence of EC50 values from a straight line when the concentration of BODIPY^-^FL GTP increases (Fig 2A, Cdc42).

Nonetheless, although the present data supports the idea that the compound targets the nucleotide binding site of the GTPases, it cannot rule out the possibility that the change in fluorescence is due to distant allosteric interactions which change the fluorescence of the nucleotide without replacement. After all, the compound poses the GTPases in an inactive state as demonstrated by cellular studies.

Importantly, we show that the compound confines GTPases to an inactive conformation as assessed by an effector binding assay. Even in the presence of growth factor stimulus, the compound was able to block downstream effector interaction and activation. Traditionally, GLISA or GST-pull down assays were used to detect active pools of GTPases [44, 45]. However, these assays suffer from being highly variable and lacking sensitivity. The assay format used here provided significantly improved sensitivity and quantitative benefits, and allowed measurement proven to be difficult with other methods [32]. Several GTPases were tested including Rab7 from the Rab subfamily, Cdc42 from the Rho subfamily, and the Ras GTPase. The results confirmed the utility of the compound for manipulating GTPase activities and tracking the consequences on known cellular pathways.

As the Rab GTPases are essential for large receptor molecule internalization and degradation [4, 38], inhibition of the Rab GTPases would be expected to delay their degradation. This was observed when cells were treated with compound **1** or its analogues. The Rho GTPases have established roles in regulating integrin activation [42, 43]. It was observed that the activation of integrin was hampered when cells were treated with compound **1** or its analogues suggesting the inhibition effects of the compounds towards Rho GTPases. Compound **1** was also shown to be inhibitory towards intracellular Ras function by decreasing viability, increasing apoptosis and mitigating the downstream Erk 1/2 signaling of a Ras-dependent cell line H358.

Although specific inhibitors are desirable for drug development, pan-inhibitors have their own uses and advantages. Molecular pathways in cells are often complex and overlapping. There are redundant pathways that could lead to downstream signal activation. Therefore, pan-inhibitors that can inhibit a group or a subgroup of proteins are usually more potent compared with specific inhibitors, and as a result are also actively pursued and studied. For example, dacomitinib is a pan-inhibitor that inhibits multiple members of the ErbB tyrosine kinase receptor family [46]. It is more effective at inhibiting the growth of head and neck cancer cell lines compared with erlotinib and cetuximab which are selective ErbB1 inhibitors. Dacomitinib is currently in a phase III clinical trial. A phosphoinositide 3-kinase (PI3K) pan-inhibitor, NVP-BKM120, can kill several oncogene addicted cell lines and is in a phase II trial [47]. For the family of histone deacetylases, the pan-inhibitor resveratrol inhibits eleven of the human histone deacetylases and has antiproliferative effects on multiple hepatoblastoma cell lines while being tolerated by primary human hepatocytes [48]. In addition, another pan-deacetylase inhibitor panobinostat showed effectiveness in inducing Hodgkin’s lymphoma cell death [49], while a pan-mTOR inhibitor aided in rhabdomyosarcoma cell apoptosis [50].

Although overexpressed GTPases and hyperactive mutants have been associated with human diseases, the discovery and development of GTPase inhibitors, whether selective or not, has had little impact compared with other families of proteins. This may be in part due to the high affinity of the associated guanine nucleotide and the globular nature of the small GTPases [9, 14]. The present study extended the investigation of guanine nucleotide competitive inhibitors [51, 52] which has been less prominent in recent years. By itself, the compound could inhibit multiple GTPases and serve as a molecular probe to block all the GTPases with overlapping functions. For comparison, a pan-inhibitor of fibroblast growth factor receptor (FGFR) helped to reveal the importance of this class of proteins in cardiovascular dysfunction while results using selective inhibitors were contradictory [53].

Nonetheless, it is necessary to consider the possible cytotoxicity arising from a pan-inhibitor. We showed that in the concentration range and time frame of our cellular assays, no significant toxicity was observed assuring that the assay results were not caused by cell death (S3 Fig), and that compound **1** is soluble and stable (S1 Text). However, after treatment for extended periods, the viability of the cells decreased particularly for a Ras-dependent cell line (Fig 10). For molecular and cellular studies with a long exposure time, cell death could be alleviated by applying the pan-inhibitor in combination with an activator of a downstream protein that facilitates cell survival, as has been described before [54, 55].

The high affinity between guanine nucleotide and GTPases makes it difficult to develop competitive inhibitors against this group of proteins. However, the compound **1** reported here not only competes with the nucleotide but also allows the GTPase to be in an inactive conformation unable to interact with downstream effectors as shown in the protein interaction studies (Fig 6). Inhibition of the GTPase-regulated pathways was further demonstrated in the phenotypic cellular assays (Figs 7–10). Nonetheless, as with any compounds lacking selectivity, it is possible that some unknown modes of action contributed to the observed results in the cellular studies. Moreover, compound **1** can serve as a template to develop more selective GTPase inhibitors. Kinase inhibitors with enhanced selectivity profiles have been developed from the pan-kinase inhibitor staurosporine [56, 57] and recently, a selective monopolar spindle 1 (Mps1) kinase inhibitor was developed by rational design from the pan-kinase inhibitor anthrapyrazolone [58]. The next step in the characterization of compound **1** will be to obtain crystal structures of complexes formed by individual GTPases and the compound. Such information will aid in structural derivatization of the compound to improve selectivity.

In conclusion, compound **1** inhibits guanine nucleotide binding to multiple GTPases as demonstrated by several biochemical, protein interaction and cellular functional assays. The compound should find utility in dissecting complex molecular pathways, mechanistic studies, and developing selective GTPase inhibitors.

## Supporting Information

S1 Fig Analogues of 1 delayed EGFR degradation in SCC-12F cells. (PDF)

S2 Fig Compound 1 and analogues in VLA-4 activation and LDV-FITC dissociation assays. (PDF)

S3 Fig Cytotoxicity of 1 and analogues. (PDF)

S1 Table Analogues of compound 1 and EC50 values. (PDF)

S1 Text Supporting Information. (PDF)
