# Supplementary material for: A Pan-GTPase Inhibitor as a Molecular Probe
Source: PLoS One. 2015 Aug 6;10(8):e0134317. doi: 10.1371/journal.pone.0134317 (PMC4527730; doi:10.1371/journal.pone.0134317)
Supplement: S1 Fig — (PDF) [file pone.0134317.s001.pdf]

S1 Fig.

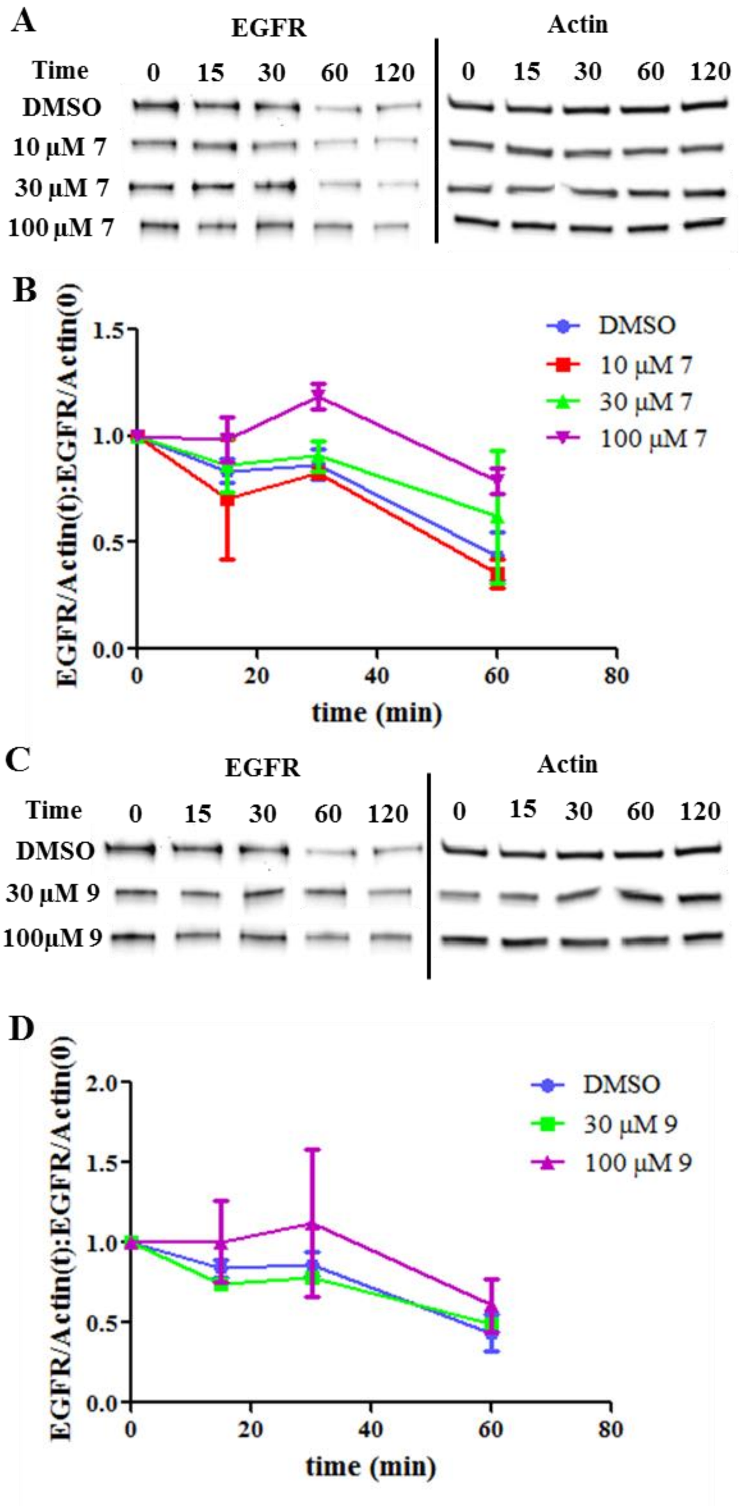
A
EGFR
Actin
Time
0
15
30
60
120
DMSO
10 µM 7
30 µM 7
100 µM 7
B
EGFR/Actin(t):EGFR/Actin(0)
time (min)
DMSO
10 µM 7
30 µM 7
100 µM 7
C
EGFR
Actin
Time
DMSO
30 µM 9
100µM 9
D
EGFR/Actin(t):EGFR/Actin(0)
time (min)
DMSO
30 µM 9
100 µM 9

S1 Fig. continued

E

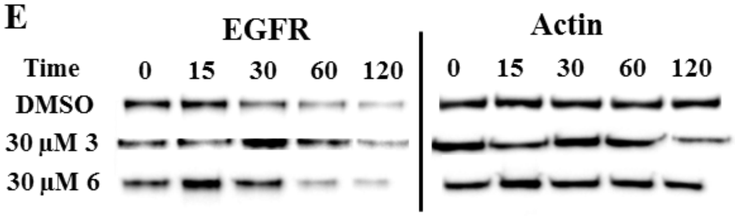

F

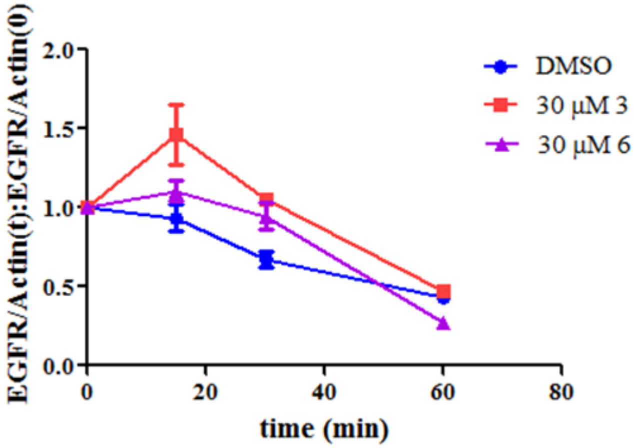

G

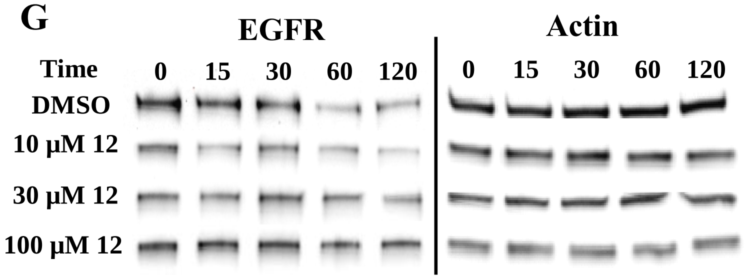

H

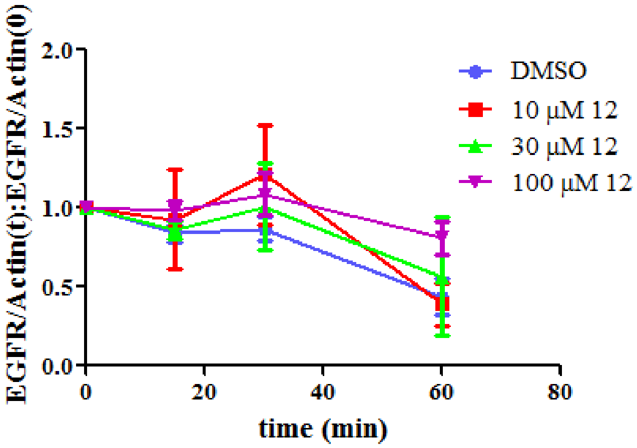

S1 Fig. Analogues of 1 delayed EGFR degradation in SCC-12F cells. A, C, E and G are representative blotting images. B, D, F, and H show the analyzed degradation time courses. Experiments for A, B, C, D, G and H were run as triplicates, while experiments for E and F were run as duplicates. Experimental details and data analysis are the same as described for Fig. 7.
