# Supplementary material for: A Pan-GTPase Inhibitor as a Molecular Probe
Source: PLoS One. 2015 Aug 6;10(8):e0134317. doi: 10.1371/journal.pone.0134317 (PMC4527730; doi:10.1371/journal.pone.0134317)
Supplement: S2 Fig — (PDF) [file pone.0134317.s002.pdf]

S2 Fig.

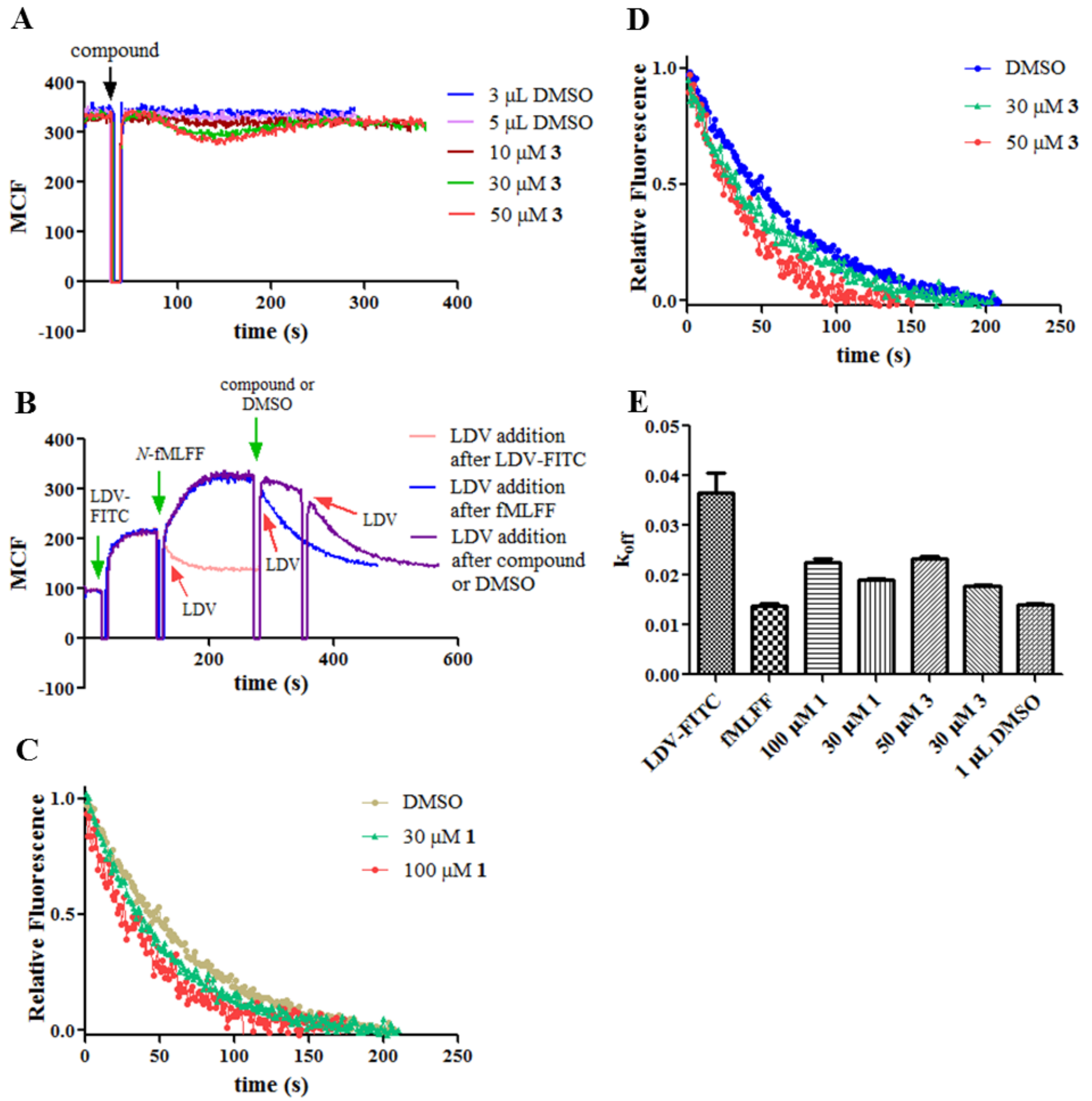

S2 Fig. Compound 1 and analogues in VLA-4 activation and LDV-FITC dissociation assays. (A) Analogues of **1** showed varied activity in the VLA-4 binding assay. Experiments were carried out as described in the main text. Fluorescence was monitored after adding compounds. Early data points were not shown. (B) A model for the dissociation rate constant determination. Non-fluorescent LDV was added to the cell suspension after either LDV-FITC, *N*- fMLFF or compounds. (C) Dissociation after inhibition by compound **1**. The decreasing fluorescence was regraphed as a ratio to the fluorescence at the time of the LDV addition. (D) Dissociation after addition of compound **3**. (E) Quantification of the dissociation rate constants according to Equation 2 in S1 Text.
