# Supplementary material for: A Pan-GTPase Inhibitor as a Molecular Probe
Source: PLoS One. 2015 Aug 6;10(8):e0134317. doi: 10.1371/journal.pone.0134317 (PMC4527730; doi:10.1371/journal.pone.0134317)
Supplement: S3 Fig — (PDF) [file pone.0134317.s003.pdf]

S3 Fig.

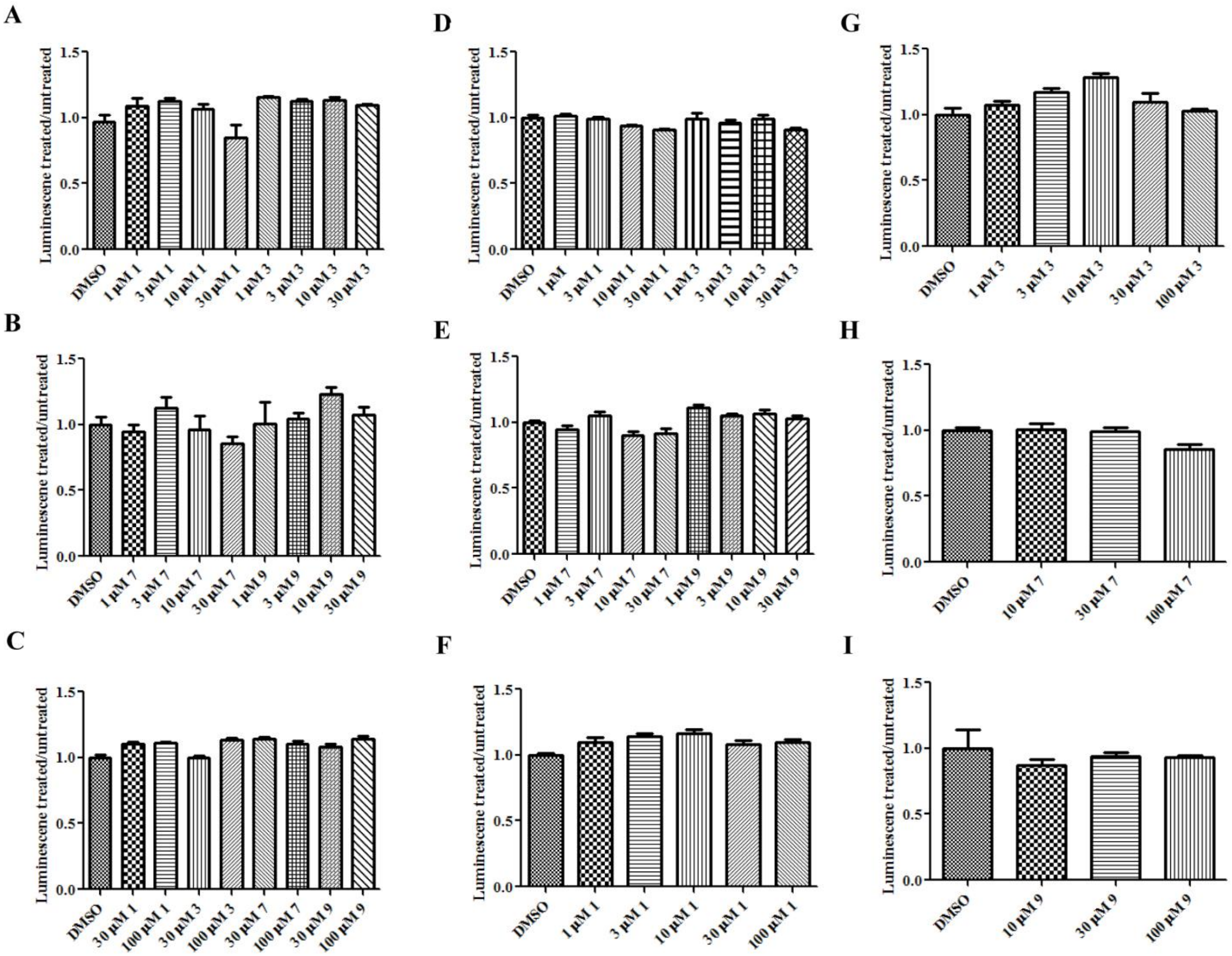

S3 Fig. Cytotoxicity of 1 and analogues. Compound **1** and its analogues did not show toxicity up to 100 µM over the experimental time frame. Compounds tested in the U937ΔST cells for 24 h (A) and (B), and for 1 h (C). Compounds tested in SCC-12F cells for 24 h (D) and (E), and for 3 h (F-I). Data are reported as ratios of luminescence to that of the DMSO treated control.
