# Supplementary material for: A Pan-GTPase Inhibitor as a Molecular Probe
Source: PLoS One. 2015 Aug 6;10(8):e0134317. doi: 10.1371/journal.pone.0134317 (PMC4527730; doi:10.1371/journal.pone.0134317)
Supplement: S1 Table — (PDF) [file pone.0134317.s004.pdf]

S1 Table

CID	structure derivation	EC_{50}						
		Rab7	H-Ras	H-Ras G12V	Cdc42	Cdc42 Q61L	RhoA	Rac1
1067700	X=O;R_1=Me;R_2=Me;R_3=OH;R_4=CS-NH-CO; R_5=phenyl	0.12 ± 0.055	0.16 ± 0.16	0.052 ± 0.044	1.09 ± 1.62	0.047 ± 0.038	0.062 ± 0.049	0.062± 0.049
1251121	R_3=OMe	>1000	>1000	>1000	>1000	>1000	NA	NA
46916263	R_1=H	0.14 ± 0.11	0.13 ± 0.10	0.13 ± 0.12	9.09 ± 12.28	4.04 ± 5.58	0.12 ± 0.07	0.08 ± 0.03
46916266	R_1=H;R_2=H	0.16 ± 0.067	0.17 ± 0.094	0.12 ± 0.12	0.11 ± 0.064	0.13 ± 0.078	0.23 ± 0.21	0.18 ± 0.13
53377401	R_2=Et	0.032 ± 0.0073	0.028 ± 0.011	0.035	2.2 ± 3.7	0.077 ± 0.087	0.046	0.053
53377399	R_1=H;R_2=t-butyl	0.47	0.14	0.13	1.57	0.94	0.20	0.14
53377403	R_1-R_2=-$(CH_2)_4$-	0.04	0.03	0.03	NA	NA	0.10	0.06
740871	R_4=CO-CH_2	>1000	>1000	>1000	>1000	>1000	NA	NA
1280844	R_4=CS-NH	NA	NA	3.55	391.00	125.70	1.39	4.06
46916265	R_4=CO-NH-CO	0.31 ± 0.09	0.44 ± 0.41	0.49 ± 0.48	17.00	0.47	0.33 ± 0.03	0.35 ± 0.05
53377397	R_5=2-MeO-phenyl	1.12	1.32	0.81	20.77	2.35	NA	NA
53301934	R_5=3-MeO-phenyl	0.15 ± 0.13	0.13 ± 0.13	0.12 ± 0.12	0.58 ± 0.83	0.50 ± 0.71	0.20 ± 0.13	0.19 ± 0.06
53301932	R_5=4-MeO-phenyl	0.14 ± 0.07	0.14 ± 0.09	0.17 ± 0.10	1.2 ± 1.0	0.23 ± 0.039	0.15 ± 0.06	0.13 ± 0.06
53301935	R_5=2-F-phenyl	0.14 ± 0.16	0.15 ± 0.18	0.12 ± 0.14	3.72 ± 5.15	0.79 ± 1.13	0.18 ± 0.22	0.18 ± 0.24
53301930	R_5=3-F-phenyl	0.32 ± 0.09	0.33 ± 0.09	0.38 ± 0.14	21.17 ± 35.48	2.20 ± 3.14	0.47 ± 0.14	0.48 ± 0.01
53377405	R_5=4-F-phenyl	0.41 ± 0.19	0.33 ± 0.22	0.091	1.0± 1.2	0.88 ± 0.47	0.11	0.15
53301931	R_5=2-Br-phenyl	0.43 ± 0.68	0.62 ± 1.00	0.43 ± 0.68	46.77 ± 66.01	14.76 ± 20.80	0.48 ± 0.74	0.60 ± 0.80
53301936	R_5=3-Br-phenyl	2.51 ± 2.10	1.72	1.08	191.81 ± 243.66	23.67 ± 23.88	>1000	31.06
53301933	R_5=4-Br-phenyl	0.30 ± 0.18	0.33 ± 0.24	0.32 ± 0.22	25.86 ± 44.07	10.89 ± 18.37	0.32 ± 0.13	0.26 ± 0.11
53377404	R_5=2-Me-phenyl	0.41	0.44	0.38	2.66	1.35	0.49	0.44
53377400	R_5=3-Me-phenyl	1.16	1.39	0.85	5.87	1.83	1.45	1.51
53377395	R_5=4-Me-phenyl	0.14	0.19	0.18	0.53	0.43	0.13	0.15
53377402	R_5=2-furyl	0.074	0.079	0.065	16.17	0.33	0.092	0.059
53377398	R_5=2-thiophene	0.025	0.035	0.024	NA	NA	0.033	0.029
53301937	X=CH_2	0.066 ± 0.017	0.067 ± 0.024	0.077 ± 0.024	0.28 ±0.16	0.17 ±0.086	0.071 ± 0.0096	0.068 ± 0.020
974814	R_3=OMe; R_4=none; R_5=none	>1000	>1000	>1000	>1000	>1000	NA	NA
1097649	R_3=OEt; R_4=CS-NH	>1000	>1000	>1000	>1000	>1000	NA	NA
46916262	R_1=H;R_2=H;R_3=$OC(CH_3)_3$	>1000	>1000	>1000	>1000	>1000	NA	NA
2810966	X=NMe;R_1=H;R_2=H;R_3=OEt; R_4=CO-NH; R_5=4-F-phenyl	>1000	>1000	>1000	>1000	>1000	NA	NA
2810963	X=NMe;R_1=H;R_2=H;R_3=OEt; R_4=CO-NH; R_5=4-Me-phenyl	>1000	>1000	>1000	>1000	>1000	NA	NA
2810964	X=NMe;R_1=H;R_2=H;R_3=OEt; R_4=CO-NH-$C(CH_3)_3$;R_5=none	>1000	>1000	>1000	>1000	>1000	NA	NA
46916261	X=NMe;R_1=H;R_2=H;R_3-R_4=-O-C(=N)-NH-;R_5=4-F-phenyl	>1000	>1000	>1000	>1000	>1000	NA	NA
46916264	X=NMe;R_1=H;R_2=H;R_3-R_4=-O-C(=N)-NH-;R_5=4-Me-phenyl	>1000	>1000	>1000	>1000	>1000	NA	NA
46916260	X=NMe;R_1=H;R_2=H;R_4=CO-NH;R_5=4-F-phenyl	>1000	>1000	>1000	>1000	>1000	NA	NA
46916259	X=NMe;R_1=H;R_2=H;R_4=CO-NH;R_5=4-Me-phenyl	49.30	30.20	32.20	26.20	28.00	NA	NA

S1 Table. Analogues of compound 1 and EC_{50} values. Analogues of compound **1** and EC_{50} values. Derivatization sites were indicated as in the main text Figure 1. For EC_{50}s with standard deviations, the values were the averages from multiple measurements. It is observed that the standard deviations for compound **1** and some of its analogs against Cdc42 are large. Therefore, the biochemical data for Cdc42 when needed for comparison is from a single set of experiments (Table 2 and Figure 2). For EC_{50}s reported without standard deviations, the values were determined from one measurement carried out in duplicate where all the data points were used to fit the sigmoidal dose-response equation. EC_{50} values were noted NA when they were not available or data did not give acceptable fit.
