# Supplementary material for: A Pan-GTPase Inhibitor as a Molecular Probe
Source: PLoS One. 2015 Aug 6;10(8):e0134317. doi: 10.1371/journal.pone.0134317 (PMC4527730; doi:10.1371/journal.pone.0134317)
Supplement: S1 Text — (PDF) [file pone.0134317.s005.pdf]

Supporting Information

FITC-LDV dissociation assay

Cytotoxicity of compound **1** and its analogues in cellular assays

Equation 1

Equation 2

Discussion on the remaining activity from the dose response assay (Figs. 1^S-4^S)

Solubility of **1** and analogues-Table 1^S

Stability of **1**-Fig. 5^S

Synthesis and characterization of **1** and some analogues (Fig. 6^S)

FITC-LDV dissociation assay

The dissociation kinetics of FITC-LDV was measured by flow cytometry following the addition of 100-fold excess of non-fluorescent LDV. (The kinetic constants obtained are the same within experimental error whether adding 100-fold or 200-fold excess non-fluorescent LDV). The fluorescence decrease was recorded (see B in S2 Fig.). For the resting state, when a fluorescence plateau was reached after the addition of LDV-FITC, LDV was added to the cell suspension. For the high affinity state, when the fluorescence reached equilibrium after the sequential addition of LDV-FITC and *N*-formyl peptide, LDV was added. To study the effect of a compound, LDV was added at the time point when the fluorescence approached a minimum. The fluorescence decrease curve was fitted to Equation 2 to obtain the dissociation rate constant k_{off}. The equilibrium binding constant K_d is the ratio of the dissociation rate constant k_{off} to the association rate constant k_{on}. Previous studies have shown that k_{on} of LDV binding to VLA-4 is largely invariable, and primarily dependent on diffusion [1]. Therefore the binding affinity can also be estimated by comparing the measured k_{off}. Treating the cells with formyl peptide stimulated the integrin to achieve the high affinity state and the k_{off} is about half of that of the resting state, i.e., 0.014 s^{-1}. Compound treatments after formyl peptide stimulation decreased the binding affinity between the integrin and the LDV-FITC peptide, causing the dissociation rate constants to be between the two values described above (see E in S2 Fig.).

*Cytotoxicity of compound **1** and its analogues in cellular assays*

Since compound **1** is a pan-GTPase inhibitor and GTPases play multiple roles in cells, it is important to confirm that any phenotypes observed in cellular assays were not due to the cytotoxicity incurred by the compound. Therefore, the cytotoxicity of compound **1** and its analogues on the viability of two cell lines U937 FPRΔST and SCC-12F, which were later used to carry out cellular assays, was examined. The CellTiter-Glo® Luminescent Cell Viability Assay Kit from Promega was used and the provided protocols were applied. Briefly, cells were added to 96-well plates. The densities of the cells were in the linear range of the luminescence change. Compounds were added at increasing concentrations (1-100 µM) and incubated for 1-24 h. After adding the luciferase substrate, the luminescence was read on a PerkinElmer VICTOR X Multilabel plate reader. The compound and its analogues did not show cytotoxicity in the experimental timeframe up to 100 µM (see S3 Fig.).

$$Inhibition\ percentage = \left(1 - \frac{remaining\ fluorescence\ after\ compound\ treatment}{fluorescence\ after\ DMSO\ treatment}\right) * \ 100\%$$

(Equation 1)

$y = A * exp(-koff * x) + \ C$ (Equation 2)

Discussion on the remaining binding activity from the dose response assay

Several experiments were conducted to study the cause of the remaining activity at high compound concentrations and its relationship if any to protein denaturation. In Fig. 4 of the main text, GTPases were incubated with the compound overnight. The compound was then removed by recovering the microsphere beads with bound GTPase by centrifugation and washing. The recovered bead-bound GTPases show identical BODIPY® FL GTP binding as the GTPases without previous compound treatment. Thus, if the compound can denature the GTPases, the protein showed the capacity to renature once the compound is removed.

Next, it was examined whether the GTPases that were used in the dose-response binding experiment were already partially denatured even before the addition of the compound. This could possibly cause nonspecific binding of BODIPY® FL GTP demonstrated as residual fluorescence. To explore this, several experiments were conducted. First, another GST tagged protein, GST-RILP was used to measure nonspecific binding of BODIPY® FL GTP to protein on beads. Also, naked beads were used to measure nonspecific binding towards beads. Secondly, after incubation with BODIPY® FL GTP and compound, the $MgCl_2$ was added to the reaction mixture to 20 mM final concentration (with no obvious pH change) in order to lock BODIPY® FL GTP that had been bound to the active site. Thirdly, after locking BODIPY® FL GTP with 20 mM $MgCl_2$, the reaction mixture was diluted 10 fold in buffer containing 20 mM $MgCl_2$ and measured. We did not observe significant nonspecific binding of BODIPY® FL GTP towards GST-RILP (Figs. 1^SA and 1^SB for raw and analyzed data, respectively). The addition of $MgCl_2$ did not decrease the remaining activity (Fig. 1^SC, compared to Fig. 1^SB). Further dilution, which is supposed to decrease non-specific binding, did not decrease remaining activity (Fig. 1^SD, compared to Fig. 1^SC). These results showed that partially denatured protein with nonspecific binding of BODIPY® FL GTP is not likely to be the reason of remaining activity.

To further test whether the GTPases were denatured by compound CID1067700, the circular dichroism (CD) spectra of the GTPases in the presence and absence of the compound were recorded. CD spectra were recorded using the Aviv Model 410 Circular Dichroism Spectrometer in a 1 mm path length rectangular cuvette at 25°C. The spectra were recorded in HEPES buffer [2] . Data points from 250 to 198 nm were recorded for Cdc42, Ras and Rab7 (1 µM) at 1 nm intervals. To examine whether CID1067700 denatures the GTPases, the proteins were incubated with the compound at different concentrations (0.5 µM, 2.5 µM and 12.5 µM) at room temperature for 30 min before CD spectra were collected. For comparison, incubation with the same volumes of DMSO was conducted in parallel. Each data point was averaged for either 1 or 4 seconds. Averaging for 4 seconds reduced the data noise but not significantly. The buffer baseline was subtracted from the GTPase spectra, while the spectrum of the compound alone in buffer was subtracted from the spectra of the GTPases with the compound. For each spectrum, three individual samples were prepared and each was scanned at least three times. The data were averaged. In spite of low resolution, a double bottom between 210 and 222 nm, which is characteristic of α-helical structures [3,4], was identifiable for all three GTPases. The presence of different concentrations of CID1067700 did not obviously change the CD spectra of the GTPases (Figs. 2^SA-C). For Ras incubated with 12.5 µM compound, the CD spectrum appeared shallower. However, the same volume of DMSO induced a similar effect (Fig. 2^SB) and DMSO is known to interfere with the CD measurement [2]. As

Fig. 1S

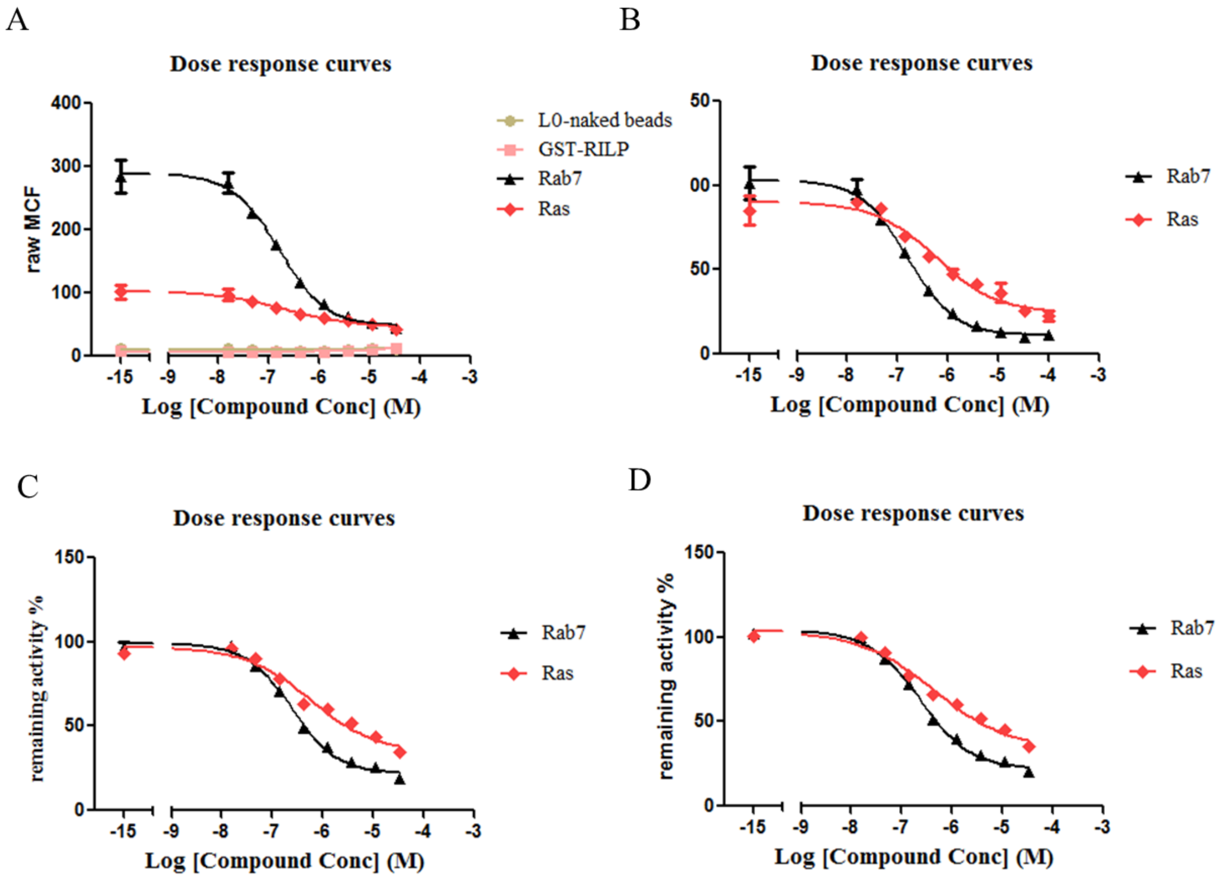

a control, 5M urea was added to the enzyme solution. The CD spectra were recorded from which the baseline of buffer containing 5 M urea was subtracted. At low wavelength, the presence of 5 M urea significantly increased the noise. Therefore, the corresponding data were not shown. The results do not support the notion that CID1067700 denatures the bulk of the protein allowing BODIPY® FL GTP to remain bound to a pool of protein. As this analysis is only to identify the extent of denaturation, the measured ellipticities were not converted to mean residual molar ellipticities.

It was then examined whether the remaining activity is due to the multiplex assay format. Two factors were considered. First, when measuring the negative control, two hundred fold excess instead of 5000 fold excess non-fluorescent GTP was used to compete with the BODIPY® FL GTP. If the negative control value is low, the calculated response percentage, which equals (sample-negative control)/(positive control-negative control)*100, may appear high causing the apparent remaining activity. However, our results showed that 200 fold and 5000 fold excess unlabeled GTP gave comparable negative control values and did not affect the response percentage (Fig. 3S). Secondly, we examined whether the multiplex setup contributed to the remaining activity. Individual GTPases were included in both single-plex and multi-plex assays. Dose response curves with similar EC50s and remaining activity were observed (Fig. 4S). These results do not support the assay format as the source of the remaining activity. Because of the availability of the reagents, Ras G12V, which has similar dose response against compound CID1067700, was used instead of Ras wild type.

Fig. 2S

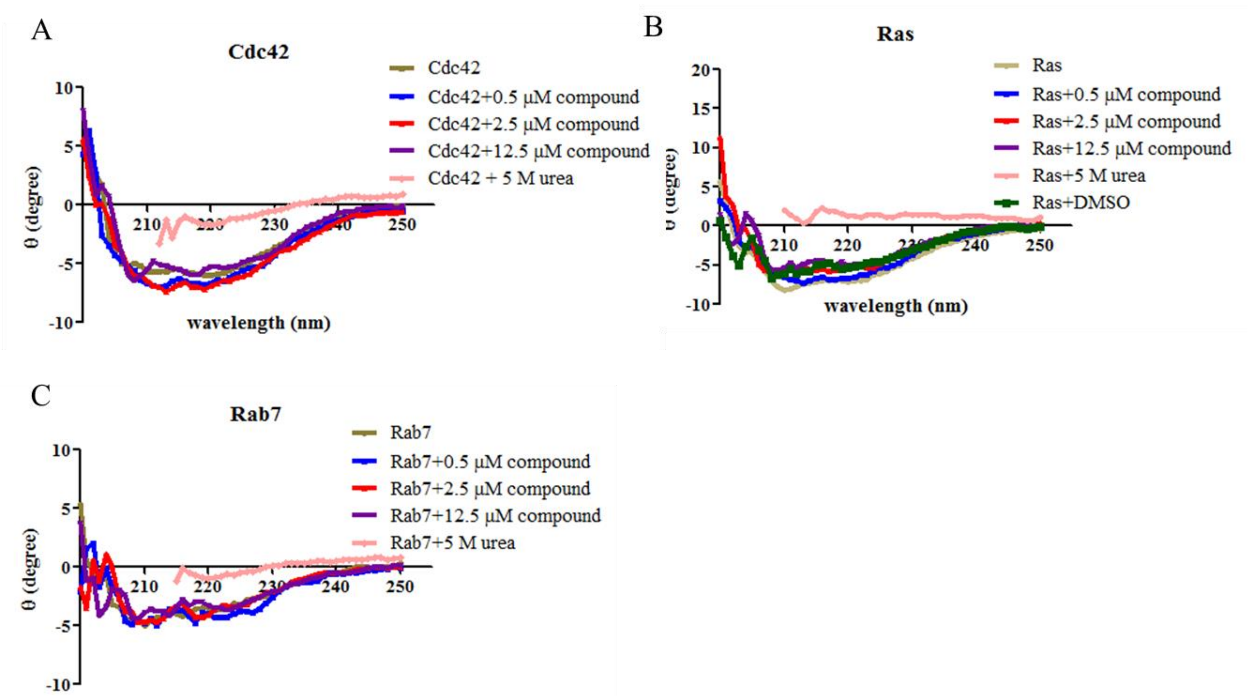
A
Cdc42
Cdc42
Cdc42+0.5 μM compound
Cdc42+2.5 μM compound
Cdc42+12.5 μM compound
Cdc42 + 5 M urea
θ (degree)
wavelength (nm)
B
Ras
Ras
Ras+0.5 μM compound
Ras+2.5 μM compound
Ras+12.5 μM compound
Ras+5 M urea
Ras+DMSO
θ (degree)
wavelength (nm)
C
Rab7
Rab7
Rab7+0.5 μM compound
Rab7+2.5 μM compound
Rab7+12.5 μM compound
Rab7+5 M urea
θ (degree)

Fig. 3S

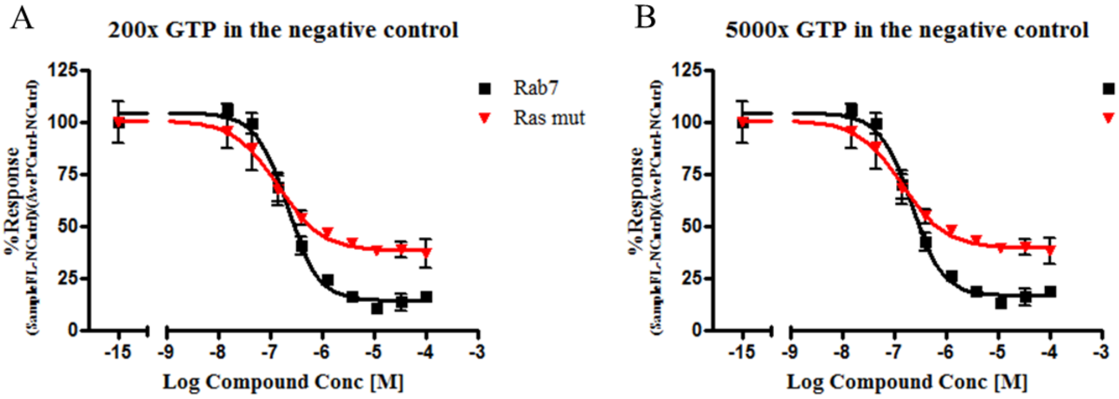
A
200x GTP in the negative control
Rab7
Ras mut
%Response
(SampleFL-NCntrl)/(AvePCntrl-NCntrl)
Log Compound Conc [M]
B
5000x GTP in the negative control
Rab7
Ras mut
%Response
(SampleFL-NCntrl)/(AvePCntrl-NCntrl)
Log Compound Conc [M]

Fig. 4[S]

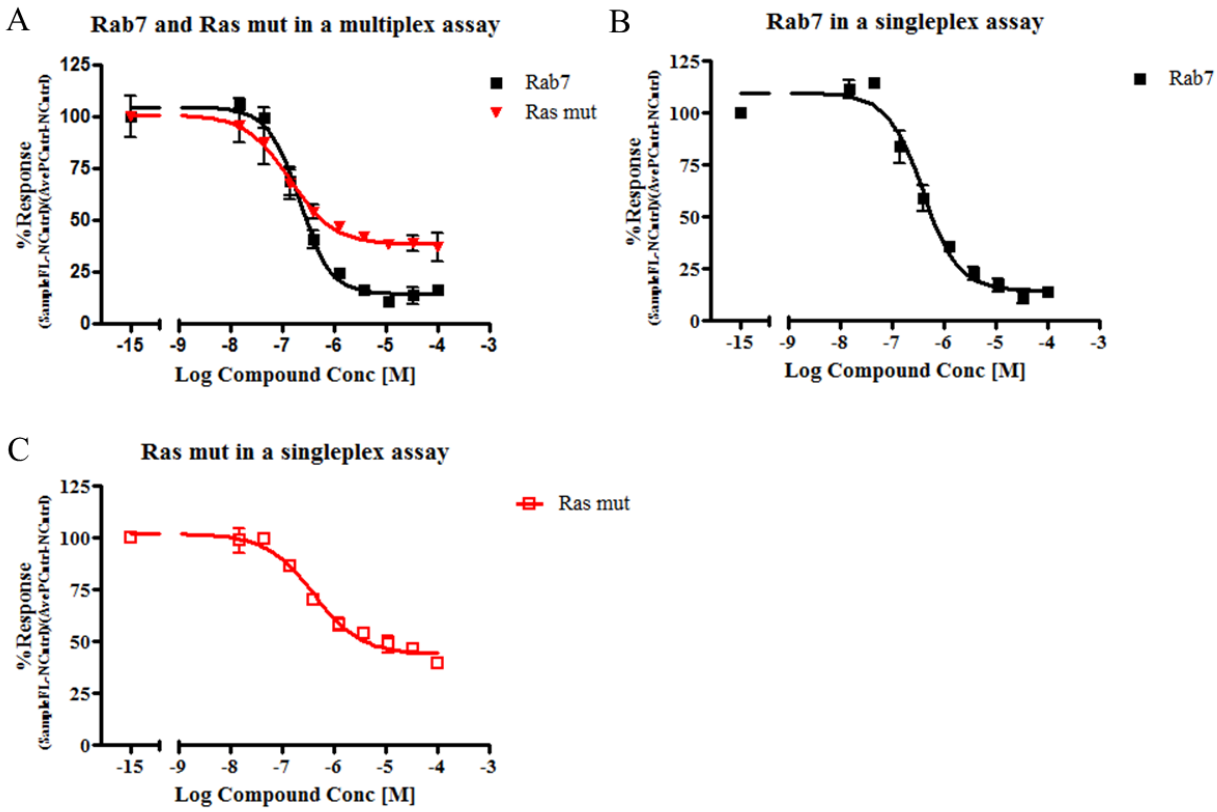

Taken together, the explanation that the remaining activity indicates deviation from competitive behavior is most consistent with all of the data. First, compound CID1067700 inhibited fluorescent GTP binding, but the inhibition is not entirely competitive. This is consistent with the effector binding assay (Fig. 6 in the manuscript) and multiple cellular functional assays (Fig. 7-10 in the manuscript) where the intracellular GTP concentration is high. It is possible that the compound blocks guanine nucleotide binding to different degrees for different GTPases, and that the incomplete blocking led to the observation of the residual fluorescence from bound BODIPY® FL GTP. The difference among the GTPases could also be seen from our multiple repetitions of the dose-dependent binding assay. Cdc42 is sensitive to assay conditions, while Ras always has higher remaining activity compared with Rab7. Exploring these differences may contribute to the identification of selective GTPase activity modulators. Secondly, as stated in the manuscript, the compound at high concentration could not induce complete dissociation of the bound fluorescent GTP (Figs. 1 and 5 in the manuscript). The EC50s obtained at different concentrations of BODIPY® FL GTP did not change in a strictly linear manner (Fig. 2 in the manuscript), which indicates deviation from competitive inhibition. Thirdly, results from the recent experiments did not support alternative explanations. Nonspecific binding of BODIPY® FL GTP to partially denatured GTPases does not likely contribute to the observed residual fluorescence (Fig. 1[S]). The presence of the compound did not denature the protein (Fig. 2[S] and Fig. 4 in the manuscript). Moreover, the assay format is not the source of the remaining fluorescence (Figs. 3[S] and 4[S]). In

conclusion, the available data support the idea that compound CID1067700 has non-classical competitive character.

Solubility of 1 and analogs:

Solubility was measured in phosphate buffered saline (PBS) at room temperature (23 °C). PBS by definition is 137 mM NaCl, 2.7 mM KCl, 10 mM sodium phosphate dibasic, 2 mM potassium phosphate [monobasic and a pH of 7.4. Detection was based on UV absorbance. Compound **1** was found to have an excellent PBS solubility measurement of > 116 µg/mL, or > 297 µM, under these conditions. Solubility was also assessed in the each of the four media used in the individual assays. Compound **1** was determined to have excellent assay media solubility, as depicted in Table 2.

Table 1^{S}. Solubility for **1** in Assay Media

compound number	CID	unit	Dose Response-buffer: 30mM HEPES, pH 7.5, 100mM KCl, 20mM NaCl, 0.01% (v/v) NP-40,	LDV-FITC assay buffer: RPMI 1640 + 10% HI-FBS	EGFR degradation assay medium: DMEM/F-12, Invitrogen # 11320-082
1	1067700	µg/mL	>68.1	> 116	> 116
		µM	>174.4	297.1	297.1
7	53301934	µg/mL	>29.7	>83	54.5
		µM	>70.6	> 197.4	129.6
9	53377405	µg/mL	>69.1	115.3	67.6
		µM	169.2	282.3	165.5
10	53301931	µg/mL	>93	>93	>93
		µM	> 198	> 198	> 198

Stability of Compound 1

Stability was measured under two distinct conditions with **1**. Stability, depicted as closed circles in the graph, was assessed at room temperature (23 °C) in PBS (no antioxidants or other protectants and DMSO concentration below 0.1%). Stability data is depicted in Fig. S8 showing the loss of compound with time over a 48 h period with a minimum of 6 time points and providing the percent remaining compound at end of the 48 h period. Under these conditions, 77% of **1** remains after 48 hours. The degradation has not been further characterized.

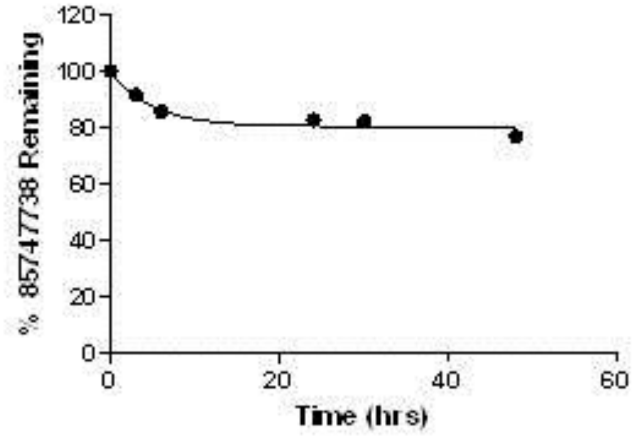

Fig. 5S. Graph depicting stability of **1** after 48 h in PBS (no additives).

To assess the chemical stability of **1** and its propensity towards nucleophilic attack, the compound was treated with a range of equivalents of L-glutathione in DMSO for 72 h at 37 °C. The three experiments were monitored by LCMS at each of the following time points: 1 h, 2 h, 4 h, 24 h, 48 h, and 72 h. *Procedure: To a solution of* ***1****, 1.0 mg, 2.56 µmol, 1 eq) in DMSO (1.0 mL) was added:*

a) L-glutathione (1.0 mg, 3.25 µmol, 1.2 eq) and the mixture stirred at 37 °C for 72 h
b) L-glutathione (1.6 mg, 5.12 µmol, 2.0 eq) and the mixture stirred at 37 °C for 72 h
c) L-glutathione (2.4 mg, 7.68 µmol, 3.0 eq) and the mixture stirred at 37 °C for 72 h

LCMS analysis of each reaction vial, taken after time (t) = 1 h, 2 h, 4 h, 24 h, 48 h, and 72 h, showed only the presence of **1**. No glutathione conjugate or other peaks were observed. These results suggest that the compound is not generally electrophilic or susceptible to protein-derived nucleophiles

General experimental and analytical details: ^{1}H and ^{13}C NMR spectra were recorded on a Bruker AM 400 spectrometer (operating at 400 and 101 MHz respectively) or a Bruker AVIII spectrometer (operating at 500 and 126 MHz respectively) in $CDCl_3$ with 0.03% TMS as an internal standard or DMSO-d_6. The chemical shifts (δ) reported are given in parts per million (ppm) and the coupling constants (J) are in Hertz (Hz). The spin multiplicities are reported as s = singlet, br. s = broad singlet, d = doublet, t = triplet, q = quartet, dd = doublet of doublet and m = multiplet. The LCMS analysis was performed on an Agilent 1200 RRL chromatograph with photodiode array UV detection and an Agilent 6224 TOF mass spectrometer. The chromatographic method utilized the following parameters: a Waters Acquity BEH C-18 2.1 x 50mm, 1.7 um column; UV detection wavelength = 214 nm; flow rate = 0.4ml/min; gradient = 5 - 100% acetonitrile over 3 minutes with a hold of 0.8 minutes at 100% acetonitrile; the aqueous mobile phase contained 0.15% ammonium hydroxide (v/v). The mass spectrometer utilized the following parameters: an Agilent multimode source which simultaneously acquires ESI+/APCI+; a reference mass solution consisting of purine and hexakis(1H, 1H, 3H-tetrafluoropropoxy) phosphazine; and a make-up solvent of 90:10:0.1 MeOH:Water:Formic Acid which was introduced to the LC flow prior to the source to assist ionization. Melting points were determined on a Stanford Research Systems OptiMelt apparatus.

2-(3-Benzoylthioureido)-5,5-dimethyl-5,7-dihydro-4H-thieno[2,3-c]pyran-3-carboxylic acid (1, CID 1067700) was purchased from ChemDiv, Inc. (CAS 314042-01-8) and purified by mass-directed reverse-phase chromatography to yield a white solid. CID1067700 can theoretically be prepared by the route described in Scheme 1 which was used to prepare its analogs.

Scheme 1

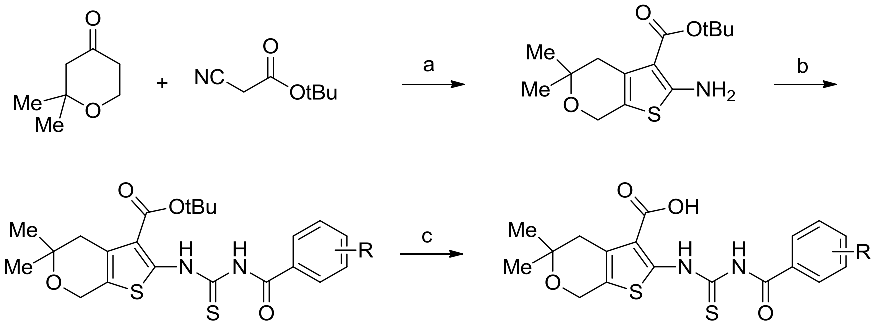

Reagents: a) morpholine, sulfur, EtOH, 50 °C; b) ArCONCS, THF, 50 °C; c) TFA, CH_2Cl_2, rt.

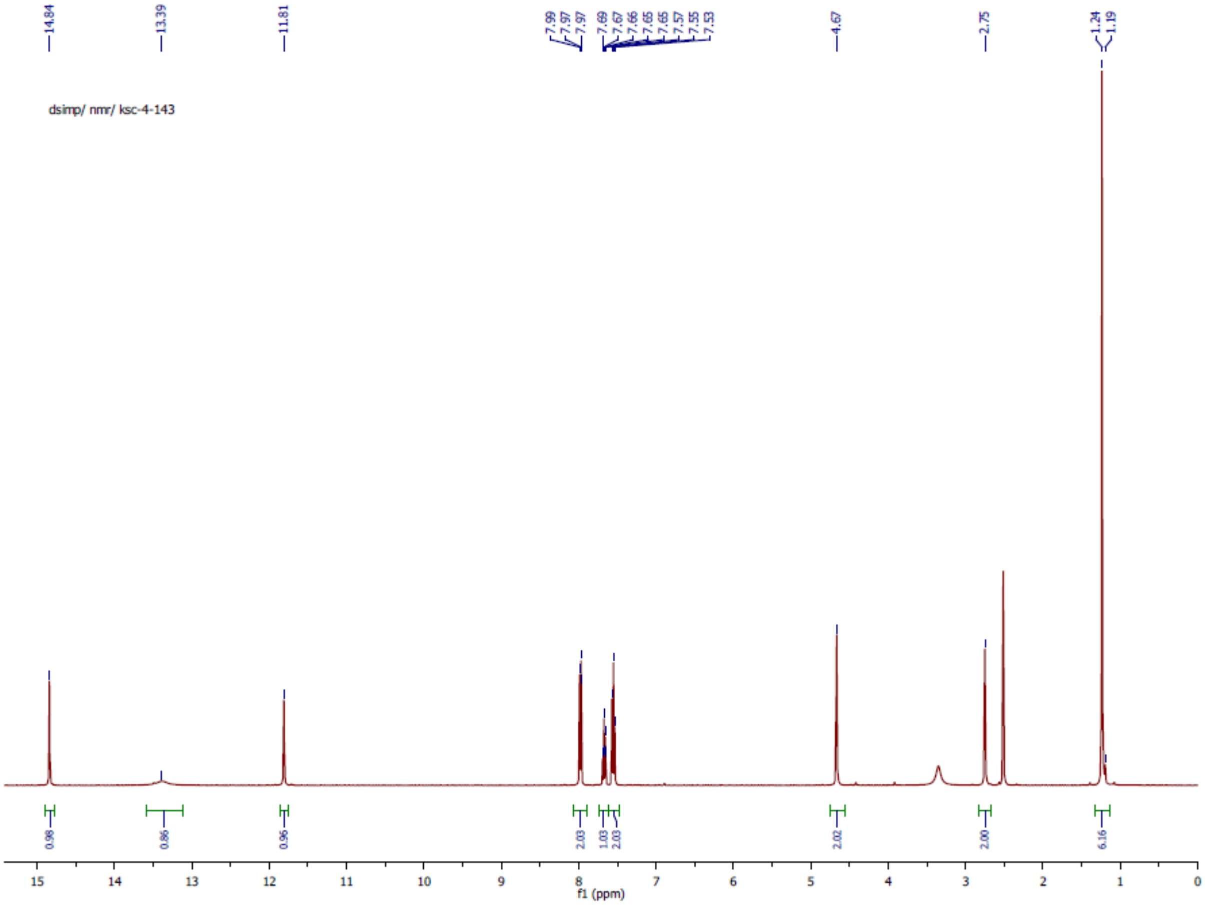

Fig. 6[S]A. Proton data for **1**, CID 1067700.

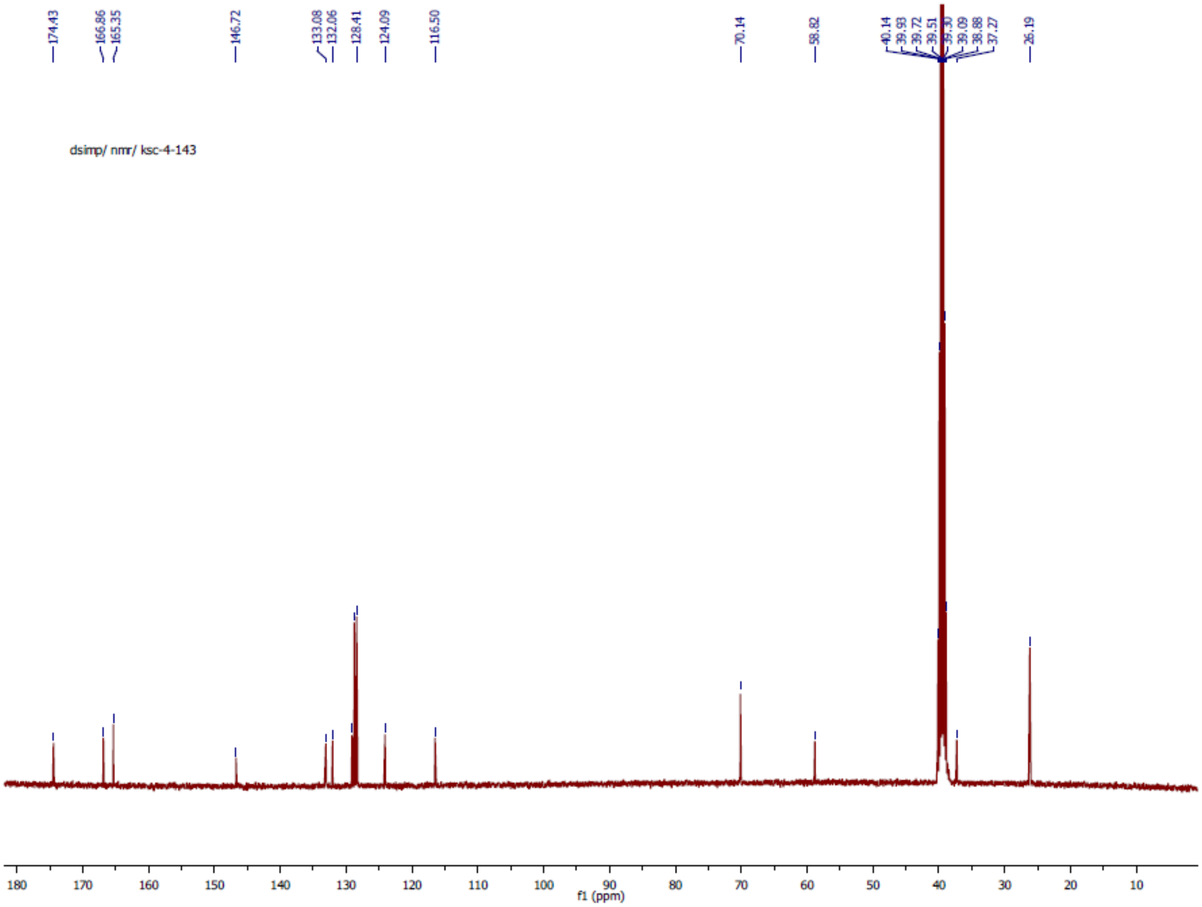

Fig. 6SB. Carbon data for **1**, CID 1067700.

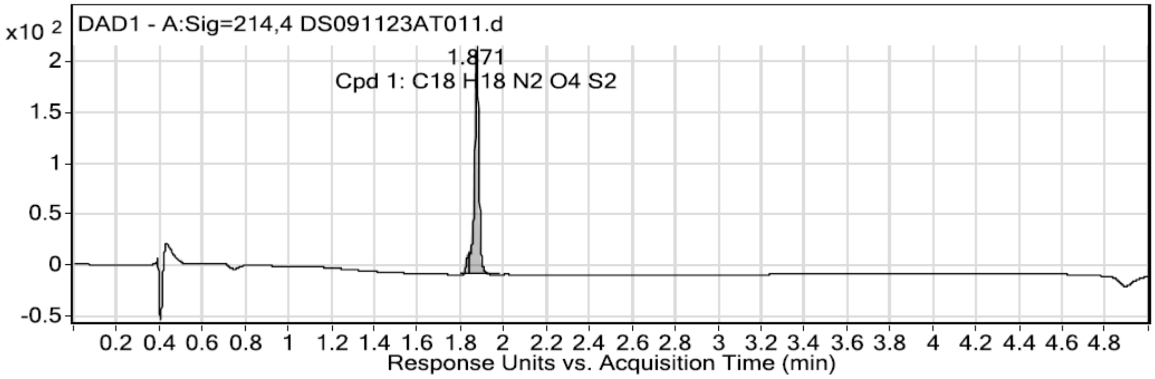

User Chromatogram Peak List

Peak #	Compound Name	RT	Height	Height %	Area	Area %	Area Sum %	Width
1		1.828	17.56	7.92	24.22	7.82	7.25	0.02
2	Cpd 1: C18 H18 N2 O4 S2	1.871	221.86	100	309.65	100	92.75	0.021

Fig. 6SC. LCMS purity data at 214 nm for **1**, CID 1067700; LCMS retention time: 1.871 min; purity at 214 nm = 92.8%.

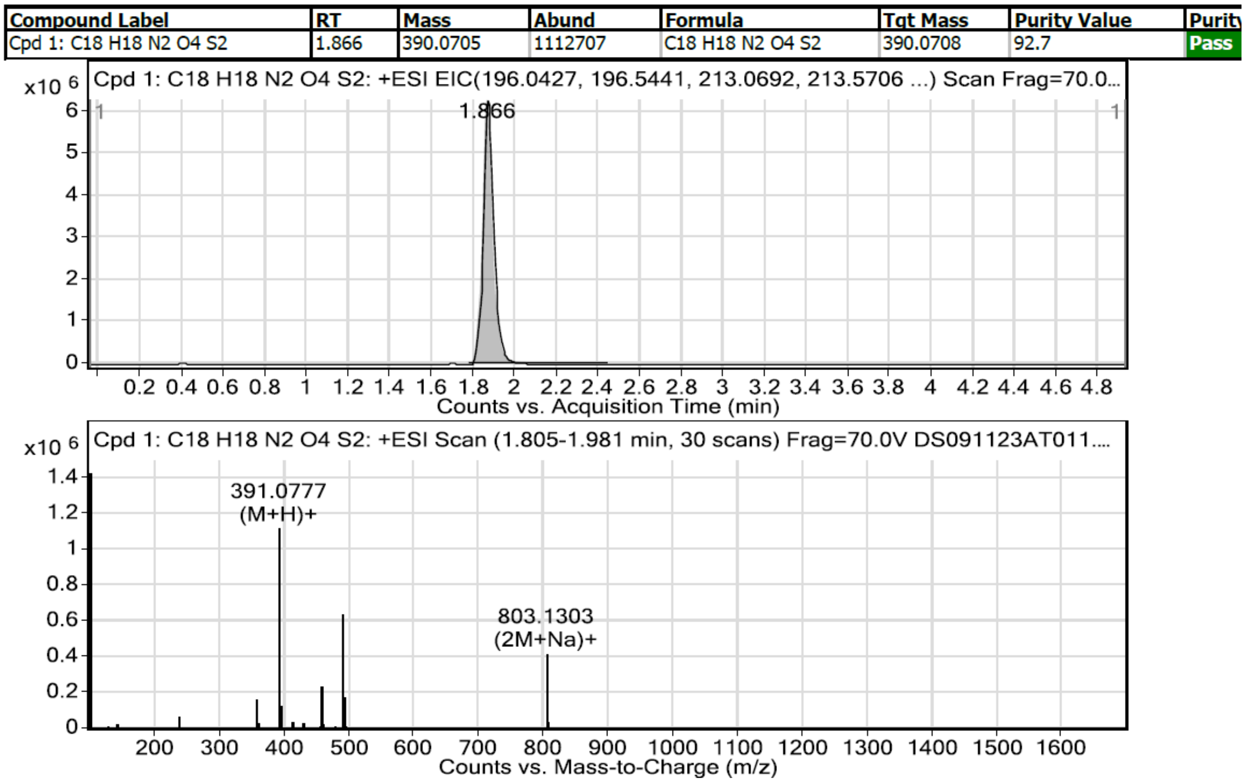

Fig. 6[S]D. HRMS data for **1**, CID 1067700: HRMS: *m/z* calculated for $C_{18}H_{19}N_2O_4S_2$ [M^++1]: 391.0781, found 391.0777.

***tert*-Butyl-2-amino-5,5-dimethyl-5,7-dihydro-*4H*-thieno[2,3-c]pyran-3-carboxylate**. Following a previously reported procedure [5], a mixture of 2,2-dimethyldihydro-2H-pyran-4(*3H*)-one (0.30 g, 2.34 mmol, 1 eq), *tert*-butyl cyanoacetate (0.37 mL, 2.57 mmol, 1.1 eq), sulfur (0.083 g, 2.57 mmol, 1.1 eq), morpholine (0.30 mL, 3.51 mmol, 1.5 eq), and ethanol (7 mL) was heated at 50 °C for 16 h. The reaction mixture was then filtered, and the filter cake washed with ethyl acetate (20 mL). Purification by silica gel chromatography (0-20% EtOAc/Hex ramp over 20 min) afforded the desired product *tert*-butyl 2-amino-5,5-dimethyl-5,7-dihydro-*4H*-thieno[2,3-c]pyran-3-carboxylate as a yellow solid (0.63 g, 2.23 mmol, 95 % yield). ^{1}H NMR (400 MHz, $CDCl_3$) δ 5.88 (s, 2H), 4.52 (apparent t, *J* = 1.9, 2H), 2.64 (apparent t, *J* = 1.9, 2H), 1.53 (s, 9H), 1.26 (s, 6H). ^{13}C NMR (101 MHz, $CDCl_3$) δ 165.22, 161.77, 130.07, 113.43, 106.94, 80.38, 70.83, 59.81, 38.72, 28.56, 26.46.

***tert*-Butyl-2-(3-(4-fluorobenzoyl)thioureido)-5,5-dimethyl-5,7-dihydro-4H-thieno[2,3-c]pyran-3-carboxylate.** *tert*-Butyl 2-amino-5,5-dimethyl-5,7-dihydro-4H-thieno[2,3-c]pyran-3-carboxylate (0.28 g, 1.00 mmol) and 4-fluorobenzoyl isothiocyanate (0.18 g, 0.99 mmol) were dissolved in THF (5 mL) and heated at 50 °C for 22 hours. The solvent was removed and EtOH (10 mL) was added. The product was filtered and rinsed with EtOH (2 x 10 mL). *tert*-Butyl 2-(3-(4-fluorobenzoyl)thioureido)-5,5-dimethyl-

5,7-dihydro-4H-thieno[2,3-c]pyran-3-carboxylate (0.330 g, 0.71 mmol, 71% yield) was isolated as a pale yellow solid. ^{1}H NMR (400 MHz, $CDCl_3$) δ 14.70 (s, 1H), 9.01 (s, 1H), 8.00 – 7.94 (m, 2H), 7.25 – 7.18 (m, 2H), 4.74 (apparent t, J = 1.5 Hz, 2H), 2.76 (apparent t, J = 1.5 Hz, 2H), 1.64 (s, 9H), 1.31 (s, 6H).

***tert*-Butyl-2-(3-(2-bromobenzoyl)thioureido)-5,5-dimethyl-5,7-dihydro-4H-thieno[2,3-c]pyran-3-carboxylate.** *tert*-Butyl 2-amino-5,5-dimethyl-5,7-dihydro-4H-thieno[2,3-c]pyran-3-carboxylate (0.28 g, 1.00 mmol) and 2-bromobenzoyl isothiocyanate (0.24 g, 1.00 mmol) were dissolved in THF (5 mL) and heated at 50 °C for 22 hours. The solvent was removed and EtOH (10 mL) was added. The product was filtered and rinsed with EtOH (2 x 10 mL). *tert*-Butyl 2-(3-(2-bromobenzoyl)thioureido)-5,5-dimethyl-5,7-dihydro-4H-thieno[2,3-c]pyran-3-carboxylate (0.26 g, 0.49 mmol, 49% yield) was isolated as a white solid. ^{1}H NMR (400 MHz, $CDCl_3$) δ 14.64 (s, 1H), 9.04 (s, 1H), 7.75 (apparent dd, J = 7.6, 1.8 Hz, 1H), 7.67 (apparent dd, J = 7.9, 1.1 Hz, 1H), 7.45 (apparent td, J = 7.5, 1.3 Hz, 1H), 7.40 (apparent td, J = 7.7, 1.9 Hz, 1H), 4.74 (apparent t, J = 1.5 Hz, 2H), 2.77 (t, J = 1.5 Hz, 2H), 1.63 (s, 9H), 1.31 (s, 6H).

***tert*-Butyl-2-(3-(3-methoxybenzoyl)thioureido)-5,5-dimethyl-5,7-dihydro-4H-thieno[2,3-c]pyran-3-carboxylate.** *tert*-Butyl 2-amino-5,5-dimethyl-5,7-dihydro-4H-thieno[2,3-c]pyran-3-carboxylate (0.28 g, 1.00 mmol) and 3-methoxybenzoyl isothiocyanate (0.20 g, 1.01 mmol) were dissolved in THF (5 mL) and heated at 50 °C for 22 hours. The solvent was removed and EtOH (10 mL) was added. The product was filtered and rinsed with EtOH (2 x 10 mL). *tert*-Butyl-2-(3-(3-methoxybenzoyl)thioureido)-5,5-dimethyl-5,7-dihydro-4H-thieno[2,3-c]pyran-3-carboxylate (0.32 g, 0.68 mmol, 68% yield) was isolated as a pale yellow solid. ^{1}H NMR (400 MHz, $CDCl_3$) δ 14.71 (s, 1H), 9.06 (s, 1H), 7.51 – 7.49 (m, 1H), 7.44 – 7.40 (m, 2H), 7.19 – 7.13 (m, 1H), 4.74 (apparent t, J = 1.5 Hz, 2H), 3.89 (s, 3H), 2.77 (apparent t, J = 1.5 Hz, 2H), 1.64 (s, 9H), 1.31 (s, 6H).

***tert*-Butyl-2-(3-(4-methoxybenzoyl)thioureido)-5,5-dimethyl-5,7-dihydro-4H-thieno[2,3-c]pyran-3-carboxylate.** A solution of *tert*-butyl 2-amino-5,5-dimethyl-5,7-dihydro-4H-thieno[2,3-c]pyran-3-carboxylate (1.0 mL, 0.083 M, 0.083 mmol) in THF was added to a solution of 4-methoxybenzoyl isothiocyanate (51 mg, 0.26 mmol) in THF (1 mL) and heated at 50 °C for 16 hours, then cooled to room temperature. The solvent was removed and EtOH (2 mL) was added. The product was filtered and rinsed with EtOH (3 x 5 mL). *tert*-Butyl 2-(3-(4-methoxybenzoyl)thioureido)-5,5-dimethyl-5,7-dihydro-4H-thieno[2,3-c]pyran-3-carboxylate (31 mg, 0.065 mmol, 78% yield) was isolated as a white solid. ^{1}H NMR (400 MHz, $CDCl_3$) δ 14.65 (s, 1H), 8.98 (s, 1H), 7.83 (apparent d, J = 8.9 Hz, 2H), 6.91 (apparent d, J = 8.9 Hz, 2H), 4.66 (s, 2H), 3.81 (s, 3H), 2.69 (s, 2H), 1.56 (s, 9H), 1.24 (s, 6H). ^{13}C NMR (101 MHz, $CDCl_3$) δ 173.90, 164.52, 164.49, 163.88, 147.40, 129.93, 129.13, 124.58, 123.65, 118.03, 114.34, 82.36, 70.79, 59.81, 55.62, 38.25, 28.49, 26.48.

***tert*-Butyl-2-(3-(2-fluorobenzoyl)thioureido)-5,5-dimethyl-5,7-dihydro-4H-thieno[2,3-c]pyran-3-carboxylate**. To a solution of *tert*-butyl 2-amino-5,5-dimethyl-5,7-dihydro-4H-thieno[2,3-c]pyran-3-carboxylate (50 mg, 0.18 mmol) in THF (3 mL) was added 2-fluorobenzoyl isothiocyanate (35.2 mg, 0.19

mmol) and the mixture heated at 60 °C for 16 h. The solvent was removed in vacuo. The residue was sonicated (1 min) with cold EtOH, filtered and the resulting solid rinsed with cold EtOH to yield *tert*-butyl 2-(3-(2-fluorobenzoyl)thioureido)-5,5-dimethyl-5,7-dihydro-4H-thieno[2,3-c]pyran-3-carboxylate (41 mg, 0.088 mmol, 50% yield) as a pale yellow solid. ^{1}H NMR (400 MHz, $CDCl_3$) δ 14.70 (s, 1H), 9.69 (s, 1H), 8.25 – 8.11 (m, 1H), 7.63 – 7.49 (m, 1H), 7.36 – 7.26 (m, 1H), 7.23 – 7.17 (m, 1H), 4.72 (s, 2H), 2.73 (d, *J* = 6.3, 2H), 1.58 (d, *J* = 2.4, 9H), 1.29 (d, *J* = 1.7, 6H). ^{13}C NMR (101 MHz, $CDCl_3$) δ 173.27, 164.59, 161.79, 161.21, 161.18, 159.30, 147.36, 135.67, 135.57, 132.79, 129.15, 125.37, 125.34, 124.64, 119.06, 118.96, 118.06, 116.70, 116.46, 82.42, 70.79, 59.81, 38.24, 28.49, 26.48.

2-(3-(4-Fluorobenzoyl)thioureido)-5,5-dimethyl-5,7-dihydro-4H-thieno[2,3-c]pyran-3-carboxylic acid (9, CID53377405). A solution of 40% v/v TFA/DCM (20 mL) was added to *tert*-butyl 2-(3-(4-fluorobenzoyl)thioureido)-5,5-dimethyl-5,7-dihydro-4H-thieno[2,3-c]pyran-3-carboxylate (0.33 g, 0.71 mmol) and the mixture was stirred at RT for 1 hour. The volatiles were removed at 30 °C and the material was purified by reverse-phase chromatography (10-100% MeCN/water). 2-(3-(4-Fluorobenzoyl)thioureido)-5,5-dimethyl-5,7-dihydro-4H-thieno[2,3-c]pyran-3-carboxylic acid (0.21 g, 0.51 mmol, 72% yield) was isolated as a pale, yellow solid. ^{1}H NMR (400 MHz, DMSO) δ 14.81 (s, 1H), 13.36 (s, 1H), 11.83 (s, 1H), 8.11 – 8.00 (m, 2H), 7.38 (apparent t, *J* = 8.9 Hz, 2H), 4.66 (s, 2H), 2.74 (s, 2H), 1.23 (s, 6H). ^{13}C NMR (101 MHz, DMSO) δ 174.33, 166.13, 165.72, 165.35, 163.63, 146.76, 131.80, 131.70, 129.08, 128.60, 128.57, 124.06, 116.48, 115.54, 115.32, 70.12, 58.80, 37.25, 26.16. LCMS retention time: 3.206 min; LCMS purity at 214 nm = 98%. HRMS *m/z* calculated for $C_{18}H_{18}FN_2O_4S_2$ [M^++1]: 409.0687, found 409.0687. Melting point 205.3 °C, decomposition.

2-(3-(2-Bromobenzoyl)thioureido)-5,5-dimethyl-5,7-dihydro-4H-thieno[2,3-c]pyran-3-carboxylic acid (10, CID53301931). A solution of 40% v/v TFA/DCM (13.4 mL) was added to tert-butyl 2-(3-(2-bromobenzoyl)thioureido)-5,5-dimethyl-5,7-dihydro-4H-thieno[2,3-c]pyran-3-carboxylate (0.25 g, 0.48 mmol) and the mixture was stirred at room temperature for 1 h. The volatiles were removed at 30 °C and the material was purified by reverse-phase chromatography (10-100% MeCN/water). 2-(3-(2-Bromobenzoyl)thioureido)-5,5-dimethyl-5,7-dihydro-4H-thieno[2,3-c]pyran-3-carboxylic acid (0.16 g, 0.33 mmol, 69% yield) was isolated as a pale, yellow solid. ^{1}H NMR (400 MHz, DMSO) δ 14.70 (s, 1H), 13.48 (s, 1H), 12.21 (s, 1H), 7.72 (apparent dd, *J* = 7.8, 1.2 Hz, 1H), 7.62 (apparent dd, *J* = 7.4, 1.8 Hz, 1H), 7.53 – 7.42 (m, 2H), 4.66 (s, 2H), 2.75 (s, 2H), 1.23 (s, 6H). ^{13}C NMR (101 MHz, DMSO) δ 174.29, 167.42, 165.95, 147.16, 137.00, 133.01, 132.56, 129.70, 129.60, 128.04, 124.63, 119.33, 116.98, 70.62, 59.29, 37.74, 26.65. LCMS retention time: 3.193 min; LCMS purity at 214 nm = 98%. HRMS *m/z* calculated for $C_{18}H_{18}BrN_2O_4S_2$ [M^++1]: 468.9886, found 468.9860. Melting point 188.4 °C, decomposition.

2-(3-(3-Methoxybenzoyl)thioureido)-5,5-dimethyl-5,7-dihydro-4H-thieno[2,3-c]pyran-3-carboxylic acid (7, CID53301934). A solution of 40% v/v TFA/DCM (20 mL) was added to tert-butyl 2-(3-(3-methoxybenzoyl)thioureido)-5,5-dimethyl-5,7-dihydro-4H-thieno[2,3-c]pyran-3-carboxylate (0.32 g, 0.67 mmol) and the mixture was stirred at room temperature for 1 h. The volatiles were removed at 30 °C and the material was purified by reverse-phase chromatography (10-100% MeCN/water). 2-(3-(3-Methoxybenzoyl)thioureido)-5,5-dimethyl-5,7-dihydro-4H-thieno[2,3-c]pyran-3-carboxylic acid (0.114 g, 0.26 mmol, 39% yield) was isolated as a pale, yellow solid. ^{1}H NMR (500 MHz, DMSO) δ 14.84 (s, 1H), 13.39 (s, 1H), 11.78 (s, 1H), 7.58 – 7.54 (m, 1H), 7.54 – 7.51 (m, 1H), 7.45 (apparent t, *J* = 7.9 Hz, 1H), 7.22 (apparent ddd, *J* = 8.3, 2.6, 0.9 Hz, 1H), 4.66 (s, 2H), 3.85 (s, 3H), 2.74 (s, 2H), 1.23 (s, 6H). ^{13}C NMR (126 MHz, DMSO) δ 174.24, 166.41, 165.25, 158.88, 146.70, 133.23, 129.51, 128.99, 123.98, 120.94, 119.29, 116.40, 113.19, 70.04, 58.71, 55.33, 37.16, 26.08. LCMS retention time: 3.199 min; LCMS purity at 214 nm = 97%. HRMS *m/z* calculated for $C_{19}H_{21}N_2O_5S_2$ $[M^{+}+1]$: 421.0886, found 421.0889. Melting point 199.3 °C, decomposition.

2-(3-(4-Methoxybenzoyl)thioureido)-5,5-dimethyl-5,7-dihydro-4H-thieno[2,3-c]pyran-3-carboxylic acid (8, CID53301932). A solution of 40% v/v TFA/DCM (2.5 mL total) was added to *tert*-butyl 2-(3-(4-methoxybenzoyl)thioureido)-5,5-dimethyl-5,7-dihydro-4H-thieno[2,3-c]pyran-3-carboxylate (34 mg, 0.071 mmol) and the mixture was stirred at room temperature for 1 h. The volatiles were removed at 30 °C and the material was purified by mass-directed reverse-phase chromatography. 2-(3-(4-Methoxybenzoyl)thioureido)-5,5-dimethyl-5,7-dihydro-4H-thieno[2,3-c]pyran-3-carboxylic acid (14 mg, 0.033 mmol, 46% yield) as a white solid. ^{1}H NMR (500 MHz, DMSO) δ 14.86 (s, 1H), 13.38 (s, 1H), 11.59 (s, 1H), 8.04 – 7.97 (m, 2H), 7.11 – 7.03 (m, 2H), 4.65 (s, 2H), 3.86 (s, 3H), 2.73 (s, 2H), 1.22 (s, 6H). ^{13}C NMR (126 MHz, DMSO) δ 174.55, 165.94, 165.28, 163.16, 146.98, 131.01, 129.02, 123.95, 123.75, 116.39, 113.76, 70.12, 58.80, 55.58, 37.25, 26.16. LCMS retention time: 3.264 min; LCMS purity at 214 nm = 99%. HRMS *m/z* calculated for $C_{19}H_{21}N_2O_5S_2$ $[M^{+}+1]$: 421.0886, found 421.0893. Melting point 213.2 °C, decomposition.

2-(3-(2-fluorobenzoyl)thioureido)-5,5-dimethyl-5,7-dihydro-4H-thieno[2,3-c]pyran-3-carboxylic acid (CID53301935). To *tert*-butyl 2-(3-(2-fluorobenzoyl)thioureido)-5,5-dimethyl-5,7-dihydro-4H-thieno[2,3-c]pyran-3-carboxylate (36 mg, 0.077 mmol) was added a solution of 2,2,2-trifluoroacetic acid (0.75 mL, 9.76 mmol) in CH_2Cl_2 (1.1 mL) [40% v/v] and the mixture stirred at RT for 1 h. The solvent was evaporated in vacuo and the residue chromatographed using reversed-phase MPLC (0-100% ACN-H_2O; 40 g C18 column) to yield 2-(3-(2-fluorobenzoyl)thioureido)-5,5-dimethyl-5,7-dihydro-4H-thieno[2,3-c]pyran-3-carboxylic acid (10.5 mg, 0.026 mmol, 33% yield) as a pale yellow solid. ^{1}H NMR (500 MHz, DMSO) δ 14.70 (s, 1H), 11.94 (s, 1H), 7.72 (apparent d, *J* = 7.3, 1H), 7.65 (s, 1H), 7.38 – 7.32 (m, 2H), 4.65 (s, 2H), 2.73 (s, 2H), 1.22 (s, 6H). ^{13}C NMR (126 MHz, DMSO) δ 173.69, 165.48, 163.91, 160.34, 158.35, 146.81, 134.19, 134.12, 130.50, 129.19, 124.64, 124.61, 124.24, 122.28, 122.17, 116.60, 116.32, 116.14, 70.20, 58.87, 37.32, 26.23. LCMS retention time: 3.275 min; LCMS purity at 214 nm = 99.6%. HRMS *m/z* calculated for $C_{18}H_{18}FN_2O_4S_2$ $[M^{+}+1]$: 409.0687, found 409.0687. Melting point 202.8 °C, decomposition.

References

1. Chigaev A, Blenc AM, Braaten JV, Kumaraswamy N, Kepley CL, et al. (2001) Real time analysis of the affinity regulation of alpha 4-integrin. The physiologically activated receptor is intermediate in affinity between resting and Mn(2+) or antibody activation. J Biol Chem 276: 48670-48678.
2. Greenfield NJ (2006) Using circular dichroism spectra to estimate protein secondary structure. Nat Protoc 1: 2876-2890.
3. Daniel H. A. Corrêa CHIR (2009) The use of circular dichroism spectroscopy to study protein folding, form and function. African Journal of Biochemistry Research 3.
4. Ramos CH, Lima MV, Jr., Silva SL, Borin PF, Regis WC, et al. (2004) Stability and folding studies of the N-domain of troponin C. Evidence for the formation of an intermediate. Arch Biochem Biophys 427: 135-142.
5. Agola JO, Hong L, Surviladze Z, Ursu O, Waller A, et al. (2012) A competitive nucleotide binding inhibitor: in vitro characterization of Rab7 GTPase inhibition. ACS Chem Biol 7: 1095-1108.
